# The Complete Chloroplast and Mitochondrial Genomes of the Green Macroalga *Ulva* sp. UNA00071828 (Ulvophyceae, Chlorophyta)

**DOI:** 10.1371/journal.pone.0121020

**Published:** 2015-04-07

**Authors:** James T. Melton, Frederik Leliaert, Ana Tronholm, Juan M. Lopez-Bautista

**Affiliations:** 1 Department of Biological Sciences, The University of Alabama, Tuscaloosa, Alabama 35487-0345, United States of America; 2 Marine Biology Research Group, Department of Biology, Ghent University, Krijgslaan 281-S8, 9000 Ghent, Belgium; 3 Smithsonian Marine Station at Fort Pierce, 701 Seaway Drive, Fort Pierce, Florida 34949, United States of America; BiK-F Biodiversity and Climate Research Center, GERMANY

## Abstract

Sequencing mitochondrial and chloroplast genomes has become an integral part in understanding the genomic machinery and the phylogenetic histories of green algae. Previously, only three chloroplast genomes (*Oltmannsiellopsis viridis*, *Pseudendoclonium akinetum*, and Bryopsis hypnoides) and two mitochondrial genomes (*O*. *viridis* and *P*. *akinetum*) from the class Ulvophyceae have been published. Here, we present the first chloroplast and mitochondrial genomes from the ecologically and economically important marine, green algal genus *Ulva*. The chloroplast genome of *Ulva* sp. was 99,983 bp in a circular-mapping molecule that lacked inverted repeats, and thus far, was the smallest ulvophycean plastid genome. This cpDNA was a highly compact, AT-rich genome that contained a total of 102 identified genes (71 protein-coding genes, 28 tRNA genes, and three ribosomal RNA genes). Additionally, five introns were annotated in four genes: *atp*A (1), *pet*B (1), *psb*B (2), and *rrl* (1). The circular-mapping mitochondrial genome of *Ulva* sp. was 73,493 bp and follows the expanded pattern also seen in other ulvophyceans and trebouxiophyceans. The *Ulva* sp. mtDNA contained 29 protein-coding genes, 25 tRNA genes, and two rRNA genes for a total of 56 identifiable genes. Ten introns were annotated in this mtDNA: *cox*1 (4), *atp*1 (1), *nad*3 (1), *nad*5 (1), and *rrs* (3). Double-cut-and-join (DCJ) values showed that organellar genomes across Chlorophyta are highly rearranged, in contrast to the highly conserved organellar genomes of the red algae (Rhodophyta). A phylogenomic investigation of 51 plastid protein-coding genes showed that Ulvophyceae is not monophyletic, and also placed Oltmannsiellopsis (Oltmannsiellopsidales) and *Tetraselmis* (Chlorodendrophyceae) closely to *Ulva* (Ulvales) and *Pseudendoclonium* (Ulothrichales).

## Introduction

The green algae are comprised of two main clades, Chlorophyta and Streptophyta. Chlorophyta includes a wide diversity of marine, freshwater and terrestrial green algae. Streptophyta includes the freshwater charophyte green algae along with the land plants [1],[2]. Chlorophyta is currently composed of the paraphyletic prasinophytes along with five classes recently coined as the “core chlorophytes” (Chlorodendrophyceae, Chlorophyceae, Pedinophyceae, Trebouxiophyceae, and Ulvophyceae) [2],[3]. Several investigations have suggested that Ulvophyceae and Trebouxiophyceae are not monophyletic and further genomic investigations with an increased taxon sampling are still needed to resolve higher taxonomic ranks [3],[4],[5],[6].

In order to help answer the phylogenetic questions that remain for the green algae and to gain a better understanding of the organellar genomic composition of these organisms, plastid and mitochondrial genomes have been sequenced throughout most of the major chlorophyte lineages, and many more organellar genomes are expected to be published in the near future. Green algae have shown to have a wide range of organellar genome sizes, content (e.g. GC%, number of genes, and number of introns), and organization of genes [2],[7],[8]. Indeed, the mitochondrial genomes (mtDNA) have ranged from as small as 13 Kbp in *Polytomella capuana* [9] to as large as 201.8 Kbp in *Chlorokybus atmophyticus* [10], and the chloroplast genomes (cpDNA) have shown even larger size differences from 37.5 Kbp in the nonphotosynthetic and parasitic *Helicosporidium* sp. [11] to 521 Kbp in *Floydiella terrestris* [12] and 525 Kbp in *Volvox carteri* [13], or possibly as big as 2 Mbp in dasycladalean green algae [14],[15]. Members of the Chlorophyceae are known to have a reduced-derived state, where most of the mitochondrial genes have been transferred to the nucleus. Conversely, mtDNAs of the Ulvophyceae and Trebouxiophyceae have shown to have an expanded pattern from the hypothesized ancestral state due to an increase of intergenic spacers and introns [8]. An expanded pattern has also been seen in the mitochondrial genomes of land plants, suggesting that convergent evolution has taken place in the two major lineages of Viridiplantae [8],[16],[17].

Ulvophyceae is well represented by marine macroalgae (known as green seaweeds) [2],[5] and contains more than 1600 species [18]. However, only three complete chloroplast genomes, *Oltmannsiellopsis viridis* [19], *Pseudendoclonium akinetum* [20], and *Bryopsis hypnoides* [21], and two mitochondrial genomes, *O*. *viridis* [17] and *P*. *akinetum* [18], have been published from organisms that are currently classified in the Ulvophyceae. Additionally, early structural data of the chloroplast genomes have been published for *Codium fragile* [15] and *Caulerpa sertulariodes* [22], and incomplete DNA sequence data has been published for *Caulerpa filiformis* [23]. It is important to point out that some of these algae could potentially belong to completely different higher taxonomic ranks in the near future. For example, Fučíková et al. [3] recovered *Oltmannsiellopsis* as sister to *Tetraselmis*, a member of the Chlorodendrophyceae and early diverging core chlorophyte, with strong support in multi-gene phylogenies mostly based on genomic plastid data.

Species of the green algal genus *Ulva* are distributed worldwide, where they are ecologically important members in marine and brackish environments and sometimes can be found in freshwater streams and lakes. Some *Ulva* species are notorious for forming harmful algal blooms called green tides in eutrophic conditions [24],[25]. These macroalgae are either monostromatic and tubular, distromatic blades, or distromatic with hollow monostromatic margins [26]. Morphology-based species level identifications of *Ulva* are notably challenging due to the lack of diagnostic morphological characters, morphological plasticity [27], and cryptic diversity [28]. There are currently 105 *Ulva* species accepted [18]. However, this number is likely to change as molecular investigations continue to reveal the true identities of these algae [24],[27],[28],[29],[30],[31],[32],[33],[34].

Transcriptomic studies have been performed on *Ulva prolifera* [35] and *U*. *linza* [36], which have helped reveal some of the genomic mechanisms that these algae have to survive in the harsh intertidal zone such as having land-plant specific genes [36]. Further, *U*. *prolifera* showed to potentially have a C_3_-C_4_ photosynthetic pathway, which could explain the ability of these species to rapidly grow during green tides [35]. However, complete organellar genomes of *Ulva* have not previously been published.

In this study, we present the first complete chloroplast and mitochondrial genomes of *Ulva*. These genomes were sequenced, assembled, and annotated as circular-mapping DNA molecules. Additionally, we compared the *Ulva* sp. genomes to the previously published chlorophyte genomes in order to gain a better understanding of the evolution of these organellar genomes. Furthermore, we performed a phylogenomic investigation to assess the monophyly of the Ulvophyceae and relationships of ulvophytes with other core chlorophytan clades.

## Materials and Methods

### Sampling and Species Identification

A monostromatic and tubular *Ulva* specimen was collected in August 2011 from a jetty near Dauphin Island Sea Lab, Alabama, USA (30º14’50.1”N, 88º04’29.1”W). Since Dauphin Island Sea Lab is a part of The University of Alabama (UA) consortium and a source for learning at several levels, no specific permission was required to collect algal samples. The specimen in this study did not involve an endangered or protected species. The specimen was preserved in silica gel, and a herbarium voucher was deposited at The University of Alabama Herbarium (UNA00071828). An image of the herbarium voucher can be viewed in Supporting Information (S1 Fig).

Due to the difficulty of identifying *Ulva* species based on morphology, species identification was based on analyses of the chloroplast-encoded *rbc*L and *tuf*A genes. These genes were selected since large amounts of data are currently available for these molecular markers. An NCBI-blastn of the genes helped find sequences with high identities. Sequences with an identity of 99% or greater were used with other previously published sequences to make alignments of *rbc*L (15 sequences; 1257 bp) and *tuf*A (12 sequences; 738 bp) using MUSCLE [37]. A neighbor-joining tree was made in Geneious R7 (http://www.geneious.com; [38]) for each alignment with the Jukes-Cantor genetic distance model and 1000 bootstrap replicates (see S2 and S3 Figs, and S1 Table in Supporting Information for gene trees and taxa used, respectively). *Monostroma grevillei* (GU183089; *rbc*L) and *Monostroma* sp. (HQ610262; *tuf*A) were used as outgroups. Additionally, the pairwise distances (bp) were calculated using MEGA 6 [39] to aid in the identification of this *Ulva* species (see S4 and S5 Figs in Supporting Information).

The *rbc*L sequence of *Ulva* sp. UNA00071828 clustered with *Ulva* sp. OTU1 from the Hawaiian Islands (GU138253; [31]). The 1257 bp alignment showed that these sequences are 100% identical, and thus, likely the same species (S4 Fig in Supporting Information). The closest *tuf*A sequence that *Ulva* sp. UNA00071828 clustered with was an unidentified *Ulva* specimen from Australia (KF195535; [40]). These sequences varied by six bp (S5 Fig in Supporting Information), and further investigations would be necessary to determine whether these organisms are conspecific or distinct entities. Due to the difficulty of identifying *Ulva* species based on morphology and the inability of relating this organism with other sequence data, we are refraining from assigning this organism a species name as O’Kelly et al. [31] also opted for. It is possible that this organism has previously been described since many *Ulva* species lack molecular data, or *Ulva* sp. UNA00071828 may represent an undescribed species. Further molecular studies in combination with morphological investigations need to be performed for an accurate identification of this *Ulva* species.

### DNA Sequencing and Assembly

Total genomic DNA was extracted using a QIAGEN DNEasy Plant Mini Kit (QIAGEN, Valencia, CA, USA). The genomic DNA was fragmented into 350 bp and sequenced using Illumina HiSeq 2000 technology at Cold Spring Harbor Laboratory, USA. The sequencing run produced ca. 23 million paired-end reads of 2 x 101 bp. Poor quality sequences and sequencing adapters were removed using Trim Galore! v0.3.7 [41], leaving 13,742,730 trimmed reads. De novo assemblies were run using Geneious, Velvet v1.2.10 [42], and CLC Genomics Workbench v6 [43] with the trimmed sequences. Following the assemblies, putative chloroplast and mitochondrial contigs were identified using a local blast in Geneious using a suite of 2,433 chloroplast and 123 mitochondrial genes from previously published chlorophyte genomes. After the contigs with blast hits for the chloroplast and mitochondrial genes were found, another de novo assembly was run separately for contigs containing chloroplast and mitochondrial genes. This resulted in one contig of ca. 100,000 bp for the chloroplast genome and another contig of ca. 73,000 bp for the mitochondrial genome. Trimmed reads were mapped iteratively to the putative chloroplast and mitochondrial contigs. This resulted in longer contigs with ca. 150 bp overlap in both contigs, which allowed the genomes to be closed.

### Annotation of the Chloroplast and Mitochondrial Genomes

Open reading frames (ORFs) of 150 bp or greater were found in Geneious using a bacterial genetic code. Protein-coding genes and rRNA genes from previous published ulvophycean chloroplast and mitochondrial genomes were then mapped to the reference chloroplast and mitochondrial contigs using the ‘Map to Reference’ option in Geneious. Annotations of the mapped genes were transferred to the reference sequence, and identities of the genes were confirmed using NCBI-blastx. Further, a blastx of other ORFs without mapped hits helped find other putative genes. A blastx hit with an E-value cut off of 10^-10^ was considered as a significant hit. To find putative tRNA genes, the chloroplast and mitochondrial contigs were submitted to tRNAscan-SE v1.21 using the default search mode and Mito/Chloroplast model [44],[45]. A comparison of tRNAs present in *Ulva* and other ulvophytes can be found in the Supporting Information (S2 and S3 Tables). Introns were also annotated and intron-exon boundaries were located by aligning the sequences of genes containing introns with those of intronless homologs. A blastx of the intronic ORFs (if present) was used to search for conserved motifs. Intron insertion sites were compared among the ulvophytes. Insertion sites of introns in protein-coding genes were determined by the nucleotide before the insertion of the intron. Insertion sites of rRNAs and tRNAs were based on alignments of 16S (NC_004431) and 23S (NC_004431) rDNA of *E*. *coli* and *trn*L(UAA) (NC_002186) of *Mesostigma viride* (NC_002186), respectively. Inverted and tandem repeats were detected using einverted and equicktandem of the EMBOSS suite [46]. Gene maps were made with OGDRAW (http://ogdraw.mpimp-golm.mpg.de/; [47]). GenBank Accession Numbers of the chloroplast and mitochondrial genomes are as follows: KP720616, KP720617, respectively.

### Rearrangements in Chlorophyte Plastid and Mitochondrial Genomes

The double-cut-and-join (DCJ) [48] genome distances were calculated with UniMoG [49],[50] to estimate the number of rearrangements between the selected genomes. The DCJ values were calculated with all genes, and a separate analysis was run without tRNAs (S4 and S5 Tables). Genome alignments with previously published chlorophyte organellar genomes were performed with the Mauve Genome Alignment v2.3.1 [51] Geneious Plugin and the progressive Mauve algorithm [52]. These alignments were made to show similarities in gene clusters called local collinear blocks (LCBs). LCBs also allow for visualizing major rearrangements as the LCBs are connected with lines in the alignment, and also display inverted regions (IR). The beginning of *rbc*L and *cox*1 were selected as the starting positions in the chloroplast and mitochondrial genomes, respectively, for the Mauve analyses (S6 and S7 Figs).

### Phylogenomic Analyses

Phylogenomic analyses were based on chloroplast genome data of 52 members of Chlorophyta, largely based on Lemieux et al. [4],[53] and Fučíková et al. [3] (S6 Table). 51 chloroplast protein-coding genes were selected from Fučíková et al. [3]: *atp*A, *atp*B, *atp*E, *atp*F, *atp*H, *atp*I, *inf*A, *pet*A, *pet*B, *pet*G, *psa*A, *psa*B, *psa*C, *psa*J, *psa*M, *psb*A, *psb*B, *psb*C, *psb*D, *psb*E, *psb*F, *psb*H, *psb*I, *psb*J, *psb*K, *psb*L, *psb*N, *psb*T, *rbc*L, *rpl*2, *rpl*5, *rpl*14, *rpl*16, *rpl*20, *rpl*23, *rpl*32, *rpl*36, *rps*3, *rps*4, *rps*7, *rps*8, *rps*9, *rps*11, *rps*12, *rps*14, *rps*18, *rps*19, *tuf*A, *ycf*3, *ycf*4 and *ycf*12. The concatenated alignment consisted of 50,595 positions and was 94% filled at the nucleotide level. Taxa with low gene coverage included *Acetabularia_acetabulum* (37 genes), *Cephaleuros parasiticus* (17 genes), *Codium decorticatum* (32 genes), *Halimeda cylindracea* (33 genes), *Tetraselmis*_sp. (11 genes), and *Trentepohlia annulata* (18 genes). We did not use the mitochondrial genome data because fewer complete mitochondrial genomes have been sequenced in the Chlorophyta and because phylogenomic analysis based on mitochondrion multigene data only weakly resolves relationships within the Chlorophyta [54].

DNA sequences were aligned for each gene separately using the ClustalW translational alignment function [55] in Geneious using a BLOSUM cost matrix, gap open penalty 10 and gap extend cost 0.1. Poorly aligned positions were removed using the Gblocks server (http://molevol.cmima.csic.es/castresana/Gblocks_server.html; [56]) using the least stringent settings: allowing smaller final blocks, gap positions within the final blocks, less strict flanking positions and many contiguous non-conserved positions. Gblocks removed 22,428 of the total 50,595 positions, leaving a nucleotide alignment of 28,167 positions. The resulting alignment was translated to obtain the amino acid alignment of 9389 positions. For the phylogenetic analysis of the DNA sequence alignment, only the first two codon positions were used.

Maximum likelihood and Bayesian trees were inferred from the DNA and amino acid (AA) alignments using RAxML v.7.3.5 [57] and MrBayes v.3.2.1 [58], respectively. For the DNA sequences, we specified a GTRCAT (RAxML) or GTR+Γ (MrBayes) model, and a partitioning strategy in which codon positions were separated (two partitions). The MrBayes analysis was run for five million (nucleotide alignment) or two million (AA alignment) generations. Two independent runs were performed for each data set. The first 10% of samples were discarded as burn-in. Convergence of the runs and stability of parameters were assessed using Tracer v.1.5 [59].

## Results and Discussion

### The Chloroplast Genome of *Ulva* sp.

**Fig 1:**
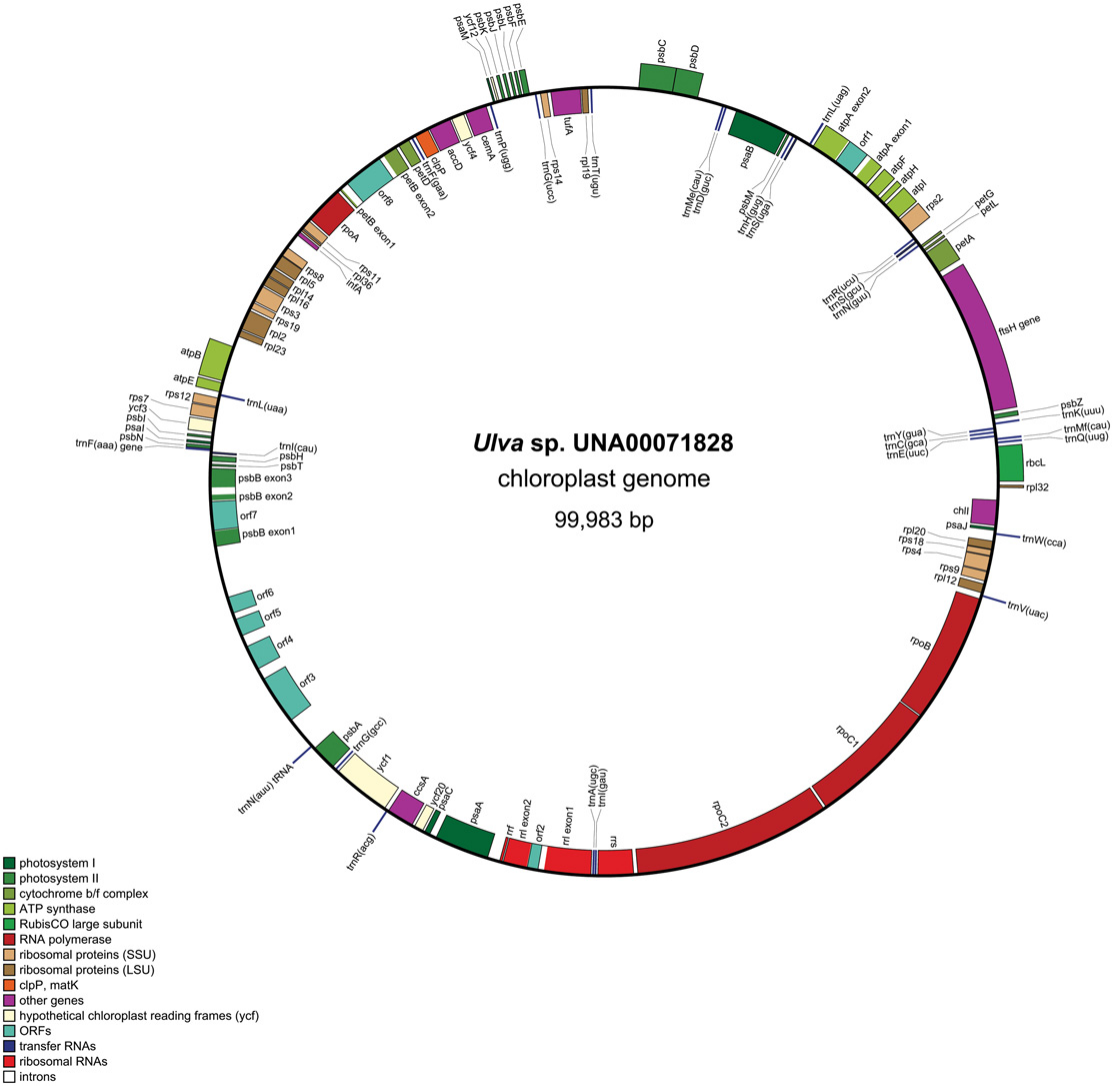
Gene map of *Ulva* sp. UNA00071828 chloroplast genome using OGDRAW. Genes in the clockwise direction are on the inside of the map, and genes in the counterclockwise direction are on the outside of the map. Annotated genes are colored according to the functional categories shown in the legend (bottom left).

A total of 1,210,556 trimmed reads combined to form a 99,983 bp circular-mapping chloroplast genome of *Ulva* sp. UNA00071828 (Fig 1). The depth of coverage at each position was a mean of 1202.2 bp, and the whole assembly had a pairwise identity of 99.8%. Table 1 compares general characteristics of the plastid genomes of *Ulva* with other core chlorophytes. To date, the chloroplast genome of *Ulva* sp. is the smallest ulvophycean chloroplast genome to be sequenced. However, this is only compared to the three complete ulvophycean genomes: *Oltmannsiellopsis viridis* (151.9 Kbp; [19]), *Pseudendoclonium akinetum* (195.8 Kbp; [20]), and *Bryopsis hypnoides* (153.4 Kbp; [21]). The percent of coding DNA (81.8%) ranks among the highest found in green algae and especially the core chlorophytes. The high coding percentage of this genome is by far the most gene compact ulvophycean genome published, compared to *O*. *viridis* (60.4%), *P*. *akinetum* (62.4%), and *B*. *hypnoides* (59.4%). The cpDNA of *Ulva* sp. ranks among the highest AT content (74.7%) compared with the core chlorophytes published thus far [2],[7]. An IR, which is commonly found in other green algae and has been found in the plastid genomes of *P*. *akinetum* [20] and *O*. *viridis* [19], was absent in this *Ulva* genome, *B*. *hypnoides* [21], and the genomes of *Codium fragile* [15] and *Caulerpa sertularioides* [22]. Thus, the *Ulva* cpDNA is not a quadripartite structure.

**Table 1:** A comparison of core chlorophyte cpDNAs.

	GenBank Accession	Genome size (Kbp)	AT (%)	Total genes^a^	Protein-coding genes	tRNA genes	rRNA genes	Free-standing ORFs^b^	Coding DNA (%)^c^	Introns^d^	Intronic ORFs	IR size (Kbp)
**Ulvophyceae**												
*Ulva* sp. UNA00071828	KP720616	100	74.7	102	71	28	3	4	81.8	5	4	none
*Pseudendoclonium akinetum*	NC_008114	195.8	62.3	105	73	29	3	12	62.4	27	19	6
*Oltmannsiellopsis viridis*	NC_008099	151.9	59.5	104	75	26	3	3	60.4	5	5	18.5
*Bryopsis hypnoides* ^e^	NC_013359	153.4	66.9	132	78	51	3	7	59.4	12	5	none
**Trebouxiophyceae**												
*Chlorella vulgaris*	NC_001865	150.6	68.4	112	76	33	3	18	54.5	3	1	none
*Parachlorella kessleri*	NC_012978	124	70	112	79	29	3	2	64.8	1	0	10.9
*Coccomyxa* sp.	NC_015084	175.7	49.3	114	79	32	3	1	45.8	1	0	none
*Leptosira terrestris*	NC_009681	195.1	72.7	107	76	28	3	1	47.3	4	1	none
**Chlorophyceae**												
*Acutodesmus obliquus*	NC_008101	161.5	73.1	99	70	26	3	1	65.1	10	7	12
*Dunaliella salina*	NC_016732	269	76.9	100	69	28	3	0	56.8	26	14	14.4
*Volvox carteri*	GU084820	461	57.6	97	66	26	5	1	23.9	8	5	16
*Gonium pectorale*	NC_020438	222.6	70.2	99	67	27	5	0	44.3	3	1	14.8
*Chlamydomonas reinhardtii*	NC_005353	203.8	65.5	99	67	27	5	2	49.9	5	0	22.2
*Oedogonium cardiacum*	NC_011031	196.5	70.5	102	70	29	3	2	77.1	23	11	35.5
*Floydiella terrestris*	NC_014346	521.2	65.5	99	69	27	3	5	27.7	30	1	none
*Schizomeris leibleinii*	NC_015645	182.8	72.8	101	68	30	3	2	71.4	37	8	none
*Stigeoclonium helveticum*	NC_008372	223.9	71.1	98	67	28	3	2	54.6	25	10	none
**Pedinophyceae**												
*Pedinomonas minor*	NC_016733	98.3	65.2	105	74	28	3	5	74.4	0	0	10.3

^a^ A sum of protein-coding genes, tRNA genes, rRNA genes. Duplicated genes only counted once

^b^ ORFs > 300 bp

^c^ % of genome consisting of conserved genes (including introns) and ORFs > 300 bp

^d^ Duplicated genes with introns only counted once

^e^ Re-annotated by Frederik Leliaert

### Gene Content

A total of 102 identifiable genes were annotated in the cpDNA of *Ulva* sp., including 71 protein-coding genes ranging from 93 bp (*psa*M) to 8067 bp (*rpo*C2), 28 tRNA genes, and three rRNA genes (*rrf*, *rrl*, and *rrs*). All of the protein-coding genes were also found in at least one of the previously published ulvophycean genome [19],[20],[21] (Table 2). The 28 tRNAs present were able to complete the full genetic code and two tRNA genes were found for *trn*G, *trn*F, *trn*I, *trn*L, *trn*M, *trn*N, *trn*R, and *trn*S (S2 Table in Supporting Information). In addition to the 102 identified genes, one freestanding ORF and three intronic ORFs had significant blastx hits in NCBI’s non-redundant protein database. The freestanding orf3 (2019 bp) had top hits of the NAD-dependent DNA ligase *Lig*A found in several *Rickettsia* species and other species of bacteria; however, with low identities (e.g. 38% identical to *R*. *montanensis*, E-value 1e^-115^, WP_014409548; 37% identical to *R*. *massiliae*, E-value 5e^-116^, WP_014365478; 37% identical to *R*. *rhipicephali*, 1e^-115^, WP_014408984).

**Table 2:** A comparison of the gene content of the *Ulva* sp. cpDNA with 17 other core chlorophytes (excluding tRNAs).

	Ulvophyceae				Trebouxiophyceae				Chlorophyceae									Ped.
	*Ulva* sp.	Pseu akin	Oltm viri	Bryo hypn	Chlo vulg	Para kess	Cocc sube	Lept terr	Acut obli	Duna sali	Volv cart	Goni pect	Chla rein	Oedo card	Floy terr	Schi leib	Stig helv	Ped mino
*acc*D	x	x	x	X	x	x	x	x	-	-	-	-	-	-	-	-	-	x
*ccs*A	x	x	x	x	x	x	x	-	x	x	x	x	x	x	x	x	x	x
*chl*B	-	-	x	x	x	x	x	x	x	x	x	x	x	x	x	x	x	-
*chl*I	x	x	x	x	x	x	x	-	-	-	-	-	-	-	-	-	-	x
*chl*L	-	-	x	x	x	x	x	x	x	x	x	x	x	x	x	x	x	-
*chl*N	-	-	x	x	x	x	x	x	x	x	x	x	x	x	x	x	x	-
*cys*A	-	-	-	x	x	x	x	x	-	-	-	-	-	-	-	-	-	x
*cys*T	-	-	-	x	x	x	x	x	-	-	-	-	-	-	-	-	-	x
*inf*A	x	x	x	x	x	x	x	x	x	-	-	-	-	x	x	-	-	-
*min*D	-	x	x	-	x	x	x	x	-	-	-	-	-	-	-	-	-	x
*pet*A	x	x	x	x	x	x	x	x	x	x	x	x	x	-	-	-	-	x
*psa*I	x	x	x	x	x	x	x	x	-	-	-	-	-	-	-	-	-	x
*psa*M	x	x	x	x	x	x	x	x	-	-	-	-	-	x	x	x	x	x
*rpl*12	x	x	x	x	x	x	x	x	x	-	-	-	-	-	-	-	-	x
*rpl*19	x	x	x	x	x	x	x	x	-	-	-	-	-	-	-	-	-	x
*rpl*32	x	x	x	x	x	x	x	x	-	-	-	-	-	x	x	x	x	x
*til*S	-	x	-	x	-	x	x	x	-	-	-	-	-	-	-	-	-	-
*ycf*12	x	x	x	x	x	x	x	-	x	x	x	x	x	x	x	x	x	x
*ycf*20	x	x	x	-	-	x	x	x	-	-	-	-	-	-	-	-	-	x
*ycf47*	-	-	-	x	-	x	x	-	-	-	-	-	-	-	-	-	-	-

Twenty-four genes (not shown in table) were shared by all 19 taxa (*atp*A, *atp*B, *atp*E, *atp*F, *atp*H, *atp*I, *cem*A, *clp*P, *fts*H, *pet*B, *pet*D, *pet*G, *pet*L, *psa*A, *psa*B, *psa*C, *psa*J, *psb*A, *psb*B, *psb*C, *psb*D, *psb*E, *psb*F, *ycf*1). Fifty-seven other genes (not shown in table) present in *Ulva* sp. were also present in most but not all of the other 18 taxa (*psb*I, *psb*J, *psb*K, *psb*L, *psb*M, *psb*N, *psb*T, *psb*Z, *rbc*L, *rpl*14, *rpl*16, *rpl*2, *rpl*20, *rpl*23, *rpl*36, *rpl*5, *rpo*A, *rpo*B, *rpo*C1, *rpo*C2, *rps*11, *rps*12, *rps*14, *rps*18, *rps*19, *rps*2, *rps*3, *rps*4, *rps*7, *rps*8, *rps*9, *tuf*A, *ycf*3, *ycf*4, *rrf*, *rrl*, *rrs*, *trn*A, *trn*C, *trn*D, *trn*E, *trn*F, *trn*G, *trn*H, *trn*I, *trn*K, *trn*L, *trn*M, *trn*N, *trn*P, *trn*Q, *trn*R, *trn*S, *trn*T, *trn*V, *trn*W, *trn*Y). An “x” represents the presence of a gene.

### Introns

Five introns were present in four genes (*atp*A, *psb*B, *pet*B, and *rrl*) of the *Ulva* chloroplast genome. *Atp*A, *rr*l, and intron 1 in *psb*B contained an intronic ORF with a LAGLIDADG conserved motif, and an intronic ORF was present in *pet*B with a reverse transcriptase conserved motif. Intronic ORFs 1 (*atp*A), 2 (*rrl*), 7 (*psb*B), and 8 (*pet*B) were 1138 bp, 767 bp, 2306 bp, and 2211 bp, respectively. An intronic ORF was not present in intron 2 in *psb*B. A total of 27, 5, and 12 introns were previously found in the chloroplast genomes of *Pseudendoclonium akinetum* [20] *Oltmannsiellopsis viridis* [19], and *Bryopsis hypnoides* [21], respectively. DNA alignments showed that the intron in *atp*A of *Ulva* is at the same position as the first intron in *P*. *akinetum*. Additionally, the intron in *rrl* of *Ulva* and the first intron in *O*. *viridis* are at homologous positions. A comparison of insertion sites of all of the ulvophyte introns can be found in Table 3.

**Table 3:** Intron insertion sites in ulvophyte mtDNA genes.

Gene	Insertion Sites	*Ulva* sp.	Pseu akin	Oltm viri	Bryo hypn
*atp*A	489	1138	1637	-	-
	513	-	344	-	-
	684	-	-	-	1005
*pet*B	69	2211	-	-	-
	534	-	-	1322	-
*psa*A	1601	-	520	-	-
	1794	-	-	-	2235
*psa*B	291	-	1368	-	-
	1047	-	1488	-	-
	1579	-	1272	-	-
*psb*A	179	-	1068	-	-
	271	-	1120	-	-
	384	-	1452	-	-
	408	-	1082	-	-
	525	-	1216	1127	-
	548	-	978	-	-
	570	-	1073	-	-
	645	-	333	-	-
	760	-	1045	-	-
	898	-	314	-	-
*psb*B	489	-	966	-	-
	600	1306	-	-	-
	689	-	-	-	1104
	772	351	-	-	-
	1022	-	1282	-	-
	1352	-	1230	-	-
*psb*C	543	-	887	-	-
	708	-	907	-	-
	882	-	960	-	-
	973	-	1051	-	-
*psb*D	740	-	1118	-	-
	1034	-	921	-	-
*rbc*L	699	-	1682	-	-
	834	-	-	-	2469
*rpl*2	326	-	-	-	837
*rpl*23	30	-	-	-	392
*rpl*5	161	-	-	-	354
*rrl*	1931	767	-	830	-
	2500	-	-	1129	-
	2593	-	816	767	-
*rrs*	510	-	-	-	971
	794	-	-	-	882
*til*S	471	-	-	-	71
*trn*L	36	-	-	-	206
*ycf*3	182	-	-	-	369

Insertion sites for protein-coding genes were based on the position relative to the homologous genes in *Mesostigma viride* (NC_002186). Insertion sites of rRNAs were based on 16S (NC_004431) and 23S (NC_004431) of *Escherichia coli*. Duplicated genes with introns are only shown once. The base pair before the intron is presented. The length of an intron (bp) is given if present at a specific site.

### The Mitochondrial Genome of ***Ulva*** sp.

**Fig 2:**
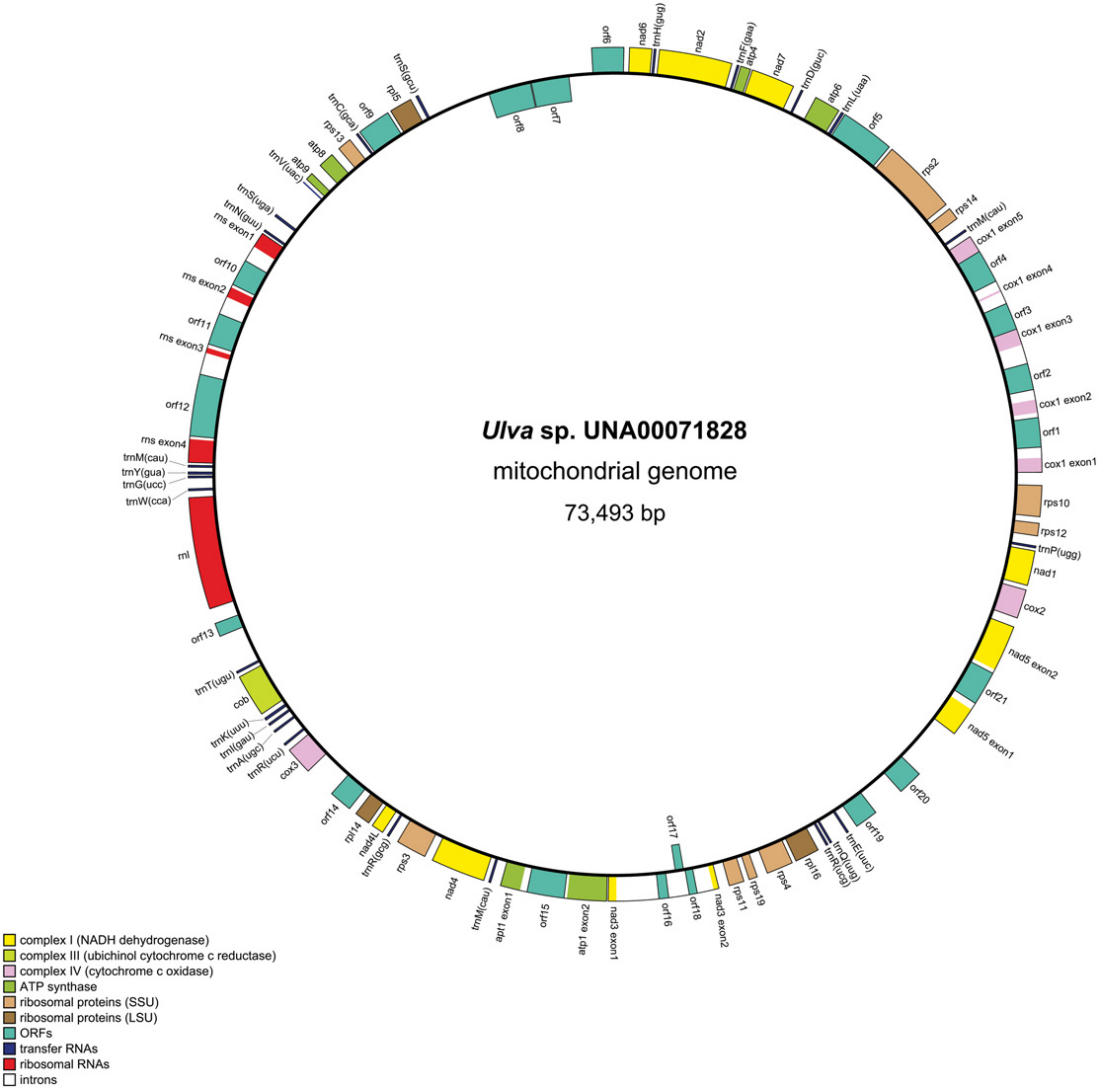
Gene map of *Ulva* sp. UNA00071828 mitochondrial genome using OGDRAW. Genes in the clockwise direction are on the inside of the map, and genes in the counterclockwise direction are on the outside of the map. Annotated genes are colored according to the functional categories shown in the legend (bottom left).

A total of 299,728 trimmed reads combined to form a 73,493 bp circular-mapping mitochondrial genome of *Ulva* sp. UNA00071828 (Fig 2). The depth of coverage at each site was a mean of 402.5 bp with a pairwise identity of 99.8%. Table 4 compares the overall characteristics of the *Ulva* sp. mtDNA with the previously published core chlorophyte mitogenomes. The mtDNA of *Ulva* sp. follows the expanded-derived pattern of the hypothesized ancestral state due to the presence of introns and/or increases in intergenic space as seen in the previously published ulvophyceans [8],[16],[17] and trebouxiophyceans [54],[60],[61],[62]. The “expanded” nature of the mtDNA has also been noted in the streptophyte lineage, suggesting the two major lineages of Viridiplantae have evolved expanded mitochondrial genomes through convergent evolution [16],[17]. However, the land plants have far larger mitochondrial genomes compared to green algae [63],[64]. Indeed, the embryophyte genomes are typically several hundred Kbp in length, and some are even over 1Mbp such as the 1.68 Mbp mtDNA of *Cucumis sativus* [65]. Thus far, all chlorophyte mtDNAs sequenced are under 100 Kbp [8]. The percent of coding DNA in the mtDNA of *Ulva* sp. was 75.8%, which is higher than the other two ulvophyceans, *P*. *akinetum* (56.7%) and *Oltmannsiellopsis viridis* (66.2%). More similar percentages of coding regions have been seen in the trebouxiophyceans, *Helicosporidium* sp. (75.7%), Trebouxiophyceae sp. MX-AZ01 (68.2%), and *Prototheca wickerhamii* (70.6%). The AT-content of the *Ulva* sp. mtDNA genome (67.6%) falls within the normal range of most green algae [8].

**Table 4:** A comparison of core chlorophyte mtDNAs.

	GenBank Accession #	Genome size (Kbp)	Conformation	AT (%)	Total genes^a^	Protein-coding genes	tRNA genes	rRNA genes	Free-standing ORFs^b^	Coding DNA (%)^c^	Introns	Intronic ORFs
**Ulvophyceae**												
*Ulva* sp. UNA00071828	KP720617	73.5	circular	67.6	56	29	25	2	9	75.8	10	12
*Oltmannsiellopsis viridis*	NC_008256	56.8	circular	66.6	54	27	24	3	5	66.2	3	4
*Pseudendoclonium akinetum*	NC_005926	95.9	circular	60.7	57	30	25	2	15	56.7	7	6
**Trebouxiophyceae**												
*Prototheca wickerhamii*	NC_001613	55.3	circular	74.2	58	29	26	3	5	70.6	5	2
*Helicosporidium* sp.	NC_017841	49.3	circular	74.4	60	32	25	3	2	75.7	3	3
Trebouxiophyceae sp.	NC_018568	74.4	circular	46.6	56	30	23	3	3	68.2	10	8
*Chlorella sorokiniana*	NC_024626	52.5	circular	66.6	60	32	25	3	1	64.1	0	0
*Coccomyxa* sp.	NC_015316	65.5	circular	46.8	59	30	26	3	0	59.1	5	1
**Chlorophyceae**												
*Polytomella magna*	NC_023091	24.4	linear	65.2	40	14	2	24	0	87.1	0	0
*Polytomella capuana*	NC_010357	13	linear	42.8	20	7	1	12	0	82	0	0
*Chlamydomonas reinhardtii*	EU306620	19	linear	55.4	23	8	3	12	0	84.2	3	3
*Chlamydomonas eugametos*	NC_001872	22.9	circular	65.4	20	7	4	9	0	84.5	9	7
*Dunaliella salina*	NC_012930	28.3	circular	69.7	19	7	3	9	0	69.7	18	2
*Gonium pectorale*	NC_020437	16	circular	61.3	23	7	4	12	0	19	1	1
*Pleodorina starrii*	NC_021108	20.4	circular	62	22	7	3	12	0	80.6	3	3
*Acutodesmus obliquus*	NC_002254	42.8	circular	63.8	47	14	27	6	4	51.9	3	2
*Bracteacoccus aerius*	NC_024755	47.2	circular	52.9	43	13	24	6	2	48	3	2
**Pedinophyceae**												
*Pedinomonas minor*	NC_000892	25.1	circular	77.8	23	11	9	3	0	60.5	1	0

^a^ A sum of protein-coding genes, tRNA genes, rRNA genes

^b^ ORFs > 300 bp

^c^ % of genome consisting of conserved genes (including introns) and ORFs > 300 bp

### Gene Content

A total of 56 identifiable genes were annotated in the mitochondrial genome, including 29 protein-coding genes ranging from 193 bp (*rps*3) to 6876 bp (*cox*1), 25 tRNA genes, and two rRNA genes (*rrs* and *rrl*). The 29 protein-coding genes were also found in at least one of the previously published ulvophycean genome (Table 5). The Ulvophyceae also have more genes in common with Trebouxiophyceae than with Chlorophyceae (Table 5), as most of the chlorophycean mitochondrial genes have been transferred to the nucleus [8]. The mtDNA of *Ulva* sp. contains 25 tRNAs that completed the full genetic code. Two *trn*L genes were present in addition to three *trn*M and three *trn*R genes. The tRNAs found in *Ulva* sp. and other ulvophyceans are shown in S3 Table in the Supporting Information. One freestanding and 12 intronic ORFs were also annotated. The freestanding orf7 (1131 bp) had a significant blastx hit as a DNA-directed RNA polymerase in the land plants *Phoenix dactylifera* (31% identical, E-value 4e^-11^, YP_005090363) and *Daucus carota* (28% identical, E-value 2e^-12^, AAS15052).

**Table 5:** A comparison of the gene content of the *Ulva* sp. mtDNA with 13 other core chlorophytes (excluding tRNAs).

	Ulvophyceae			Trebouxiophyceae				Chlorophyceae								Ped.
	*Ulva* sp.	Pseuakin	Oltm viri	Prot wick	Heli sp.	Treb sp.	Chlo soro	Cocc sp.	Poly magn	Chla rein	Duna sali	Goni pect	Pleo star	Acut obli	Brac mino	Pedi mino
*apt*1	x	x	x	x	x	x	x	x	-	-	-	-	-	-	-	-
*atp*4	x	x	x	x	x	x	x	x	-	-	-	-	-	-	-	-
*atp*6	x	x	x	x	x	x	x	x	-	-	-	-	-	x	x	x
*atp*8	x	x	x	x	x	x	x	x	-	-	-	-	-	-	-	x
*atp*9	x	x	x	x	x	x	x	x	-	-	-	-	-	x	x	-
*cox*2	x	x	x	x	x	x	x	x	-	-	-	-	-	x	x	-
*cox*3	x	x	x	x	x	x	x	x	-	-	-	-	-	x	x	-
*nad*3	x	x	x	x	x	x	x	x	-	-	-	-	-	x	x	x
*nad*4L	x	x	x	x	x	x	x	x	-	-	-	-	-	x	x	x
*nad*7	x	x	x	x	x	x	x	x	-	-	-	-	-	-	-	-
*nad*9	-	-	x	x	x	x	x	x	-	-	-	-	-	-	-	-
*rps*2	x	x	x	x	x	x	x	x	-	-	-	-	-	-	-	-
*rps*3	x	x	x	x	x	x	x	x	-	-	-	-	-	-	-	-
*rps*4	x	x	-	x	x	x	x	x	-	-	-	-	-	-	-	-
*rps*7	-	-	-	x	x	x	x	x	-	-	-	-	-	-	-	-
*rps*10	x	x	-	x	x	x	x	x	-	-	-	-	-	-	-	-
*rps*11	x	x	x	x	x	-	x	-	-	-	-	-	-	-	-	-
*rps*12	x	x	x	x	x	x	x	x	-	-	-	-	-	-	-	-
*rps*13	x	x	x	x	x	x	x	x	-	-	-	-	-	-	-	-
*rps*14	x	x	x	x	x	x	x	x	-	-	-	-	-	-	-	-
*rps*19	x	x	x	x	x	x	x	x	-	-	-	-	-	-	-	-
*rpl*2	-	-	x	-	-	-	-	-	-	-	-	-	-	-	-	-
*rpl*5	x	x	-	x	x	x	x	x	-	-	-	-	-	-	-	-
*rpl*6	-	-	-	x	x	-	x	-	-	-	-	-	-	-	-	-
*rpl*14	x	x	-	-	-	-	-	-	-	-	-	-	-	-	-	-
*rpl*16	x	x	x	x	x	x	x	x	-	-	-	-	-	-	-	-
*rrn*5	-	-	x	x	x	x	-	x	-	-	-	-	-	-	-	-
*tat*C	-	x	x	-	x	x	x	-	-	-	-	-	-	-	-	-

Nine genes (not shown in table) were present in all core chlorophytes (*nad*1, *nad*2, *nad*4, *nad*5, *nad*6, *cob*, *cox*1, *rrs*, *rrl*). An “x” represents the presence of a gene.

### Introns

A total of 10 introns were present in 5 genes (*atp*1, *cox*1, *nad*3, *nad*5, and *rrs*) in the mitochondrial genome of *Ulva* sp. Multiple introns were present in *cox*1 (4) and *rrs* (3). The presence of introns in *cox*1 indeed confirms why amplifying this gene in PCR has been such a difficult task [32]. Intronic ORFs with a LAGLIDADG conserved motif were found in *atp*1 (orf15: 1345 bp), *nad*5 (orf21: 1309 bp), each of the four introns in *cox*1 (orf1: 1287 bp, orf2: 1590 bp, orf3: 1162 bp, and orf4: 1240 bp, respectively), and intron 1 and intron 2 in *rrs* (orf10: 1319 bp and orf11: 1534 bp, respectively). Intron 3 in *rrs* contained an intronic ORF (orf12: 1734 bp) with a reverse transcriptase conserved motif. The intron in *nad*3 had two intronic ORFs (orf16: 288 bp; orf17: 156 bp) with a reverse transcriptase conserved motif and 1 intronic ORF (orf18: 240 bp) with a group II maturase conserved motif (GIIM). A total of 7 were previously annotated in the mtDNA of *Pseudendoclonium akinetum* [16] and 3 introns in *Oltmannsiellopsis viridis* [17]. DNA alignments showed that *Ulva* sp. and *P*. *akinetum* have a few introns at homologous positions. These include the intron in *apt*1, introns 1 and 2 in *cox*1, and intron 3 in *Ulva* and intron 4 in *P*. *akinetum*. Intron insertion sites in the ulvophyte mtDNAs can be found in Table 6.

**Table 6:** Intron insertion sites of ulvophyte mtDNA genes.

Gene	Insertion Sites	*Ulva* sp.	Pseu akin	Oltm viri
*atp*1	540	1345	1424	-
*cob*	474	-	-	1226
	865	-	911	-
*cox*1	429	1287	2048	-
	751	1590	1595	-
	773	-	1659	-
	781	-	-	2414
	1149	1162	1731	-
	1167	1240	-	-
*nad*3	218	2727	-	-
*nad*5	664	1309	-	-
*rrs*	521	1319	-	-
	801	1534	-	-
	915	2449	-	-
*rrl*	2500	-	-	1258

Insertion sites for protein-coding genes were based on the position relative to the homologous genes in *Mesostigma viride* (NC_002186). Insertion sites of rRNAs were based on 16S (NC_004431) and 23S (NC_002186) of *Escherichia coli*. The base pair before the inserted intron is given. If an intron was present at a specific site, the length of the intron (bp) is given if present.

### Rearrangements in the Chlorophyte cpDNA and mtDNA

The number of rearrangements between organisms from lineages throughout Chlorophyta was estimated by calculating the double-cut-and-join (DCJ) values. The DCJ values calculated for the cpDNAs and mtDNAs with tRNAs are shown in Tables 7 and 8, respectively. The DCJ values calculated for cpDNAs and mtDNAs without tRNAs can be found in the Supporting Information (S4 and S5 Tables, respectively). Mauve alignments of the selected chlorophyte genomes can also be found in the Supporting Information (S6 and S7 Figs for cpDNA and mtDNA, respectively).

**Table 7:** DCJ values for chlorophyte cpDNAs of chlorophytes (includes tRNAs).

	*Ulva* sp.	Pseu akin	Oltm viri	Bryo hypn	Chlo vulg	Cocc sp.	Acut obli	Duna sali	Oedo card	Pedi mino	Pycn prov
*Ulva* sp.	0	37	58	63	55	64	76	74	76	57	57
*Pseu akin*	-	0	59	67	58	67	75	72	78	56	56
*Oltm viri*	-	-	0	65	56	66	75	78	81	54	58
*Bryo hypn*	-	-	-	0	67	73	78	79	84	65	61
*Chlo vulg*	-	-	-	-	0	62	74	69	76	47	57
*Cocc* sp.	-	-	-	-	-	0	77	75	79	58	60
*Acut obli*	-	-	-	-	-	-	0	55	76	69	72
*Duna sali*	-	-	-	-	-	-	-	0	72	68	71
*Oedo card*	-	-	-	-	-	-	-	-	0	75	76
*Pedi mino*	-	-	-	-	-	-	-	-	-	0	58
*Pycn prov*	-	-	-	-	-	-	-	-	-	-	0

The GenBank accession numbers used are as follows: *Ulva* sp. (KP720616), *Pseudendoclonium akinetum* (NC_008114), *Oltmannsiellopsis viridis* (NC_008099), *Bryopsis hypnoides* (NC_013359), *Chlorella vulgaris* (NC_001865), *Coccomyxa* sp. C-169 (NC_015084), *Acutodesmus obliquus* (NC_008101), *Dunaliella salina* (NC_016732), *Oedogonium cardiacum* (NC_011031), *Pedinomonas minor* (NC_016733), *Pycnococcus provasolii* (NC_012097).

**Table 8:** DCJ values calculated for mtDNAs of chlorophytes (includes tRNAs).

	*Ulva* sp.	Pseu akin	Oltm viri	Prot wick	Heli sp.	Chlo vari	Acut obli	Pedi mino	Neph oliv	Ostr taur	Pycn prov
*Ulva* sp.	0	36	39	47	47	47	27	16	48	44	32
*Pseu akin*	-	0	45	44	48	45	29	16	49	44	32
*Oltm viri*	-	-	0	47	46	45	26	16	46	38	28
*Prot wick*	-	-	-	0	23	27	27	16	46	42	31
*Heli* sp.	-	-	-	-	0	27	28	17	50	45	31
*Chlo vari*	-	-	-	-	-	0	31	15	45	45	28
*Acut obli*	-	-	-	-	-	-	0	11	31	28	22
*Pedi mino*	-	-	-	-	-	-	-	0	15	14	12
*Neph oliv*	-	-	-	-	-	-	-	-	0	24	30
*Ostr taur*	-	-	-	-	-	-	-	-	-	0	26
*Pycn prov*	-	-	-	-	-	-	-	-	-	-	0

The GenBank accession numbers used are as follows: *Ulva* sp. (KP720617), *Pseudendoclonium akinetum* (NC_005926), *Oltmannsiellopsis viridis* (NC_008256), *Prototheca wickerhamii* (NC_001613), *Helicosporidium* sp. (NC_017841), *Chlorella variabilis* (NC_025413), *Acutodesmus obliquus* (NC_002254), *Pedinomonas minor* (NC_000892), *Nephroselmis olivacea* (NC_008239), *Ostreococcus tauri* (NC_008290), *Pycnococcus provasolii* (NC_013935).

The DCJ values and Mauve alignments show that both organellar genomes of chlorophytes are highly rearranged. This is in contrast to the highly conserved organellar genomes of red algae (Rhodophyta) [66],[67]. The cpDNA of *Ulva* had the lowest DCJ value with *Pseudendoclonium* (37 with tRNAs and 22 without tRNAs), which was expected since these organisms are more closely related than the other organisms in this analysis. Of the expanded mtDNAs (i.e. Ulvophyceae and Trebouxiophyceae), *Ulva* also had the lowest DCJ value with *Pseudendoclonium* (36 with tRNAs and 17 without tRNAs). The lower DCJ values of *Ulva* compared to *Pedinomonas*, *Acutodesmus*, and *Pycnococcus* according to Table 8 and S7 Table are likely due to the reduced gene count in the mtDNA of these three organisms.

### Phylogenomic Analyses

**Fig 3:**
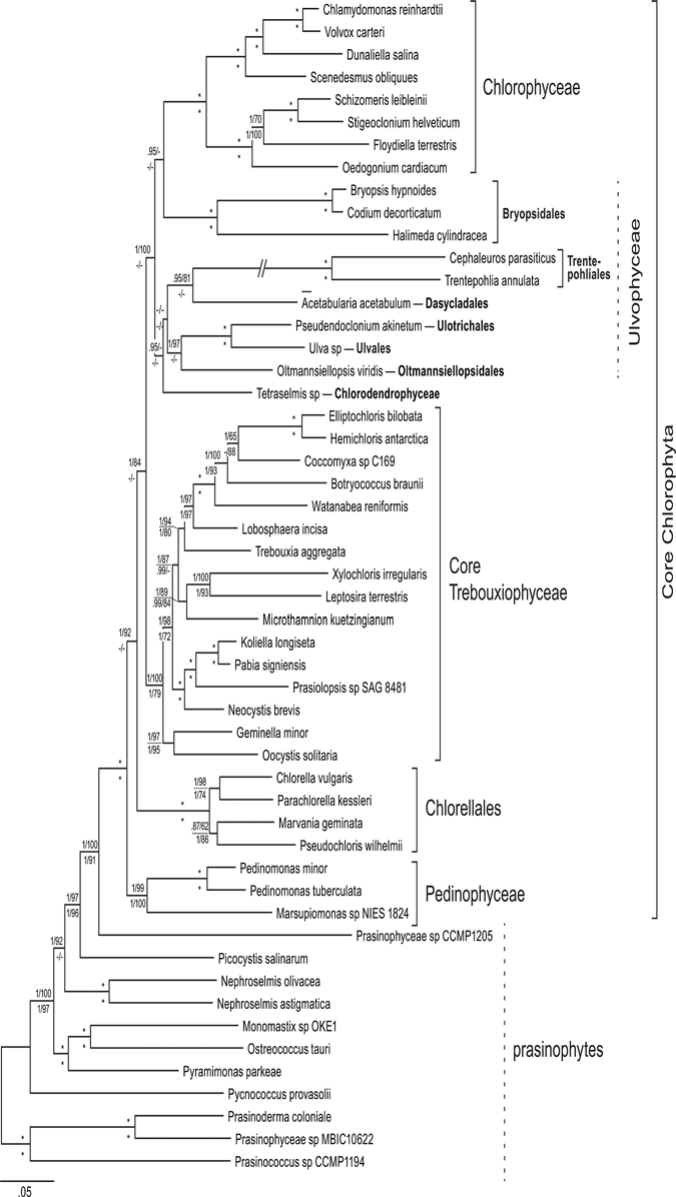
Bayesian majority rule tree showing all compatible partitions, inference from the nucleotide alignment of 51 concatenated chloroplast genes (first two codon positions: 28,167 nucleotide positions and 52 terminal taxa). Node support is given as Bayesian posterior probabilities and maximum-likelihood (ML) bootstrap values of the nucleotide analyses (above branches), and the amino acid analyses (below branches); values <0.9 and 50, respectively, are not shown; asterisks indicated full support in both the Bayesian and ML analyses.

The phylogenetic tree resulting from Bayesian analyses of the DNA alignment, along with branch support from the other analyses (ML and BI of the DNA and AA alignments) is presented in Fig 3. The ML and Bayesian trees based on an amino acid alignment are presented in the Supporting Information (S8 and S9 Figs, respectively).

As expected, *Ulva* (Ulvales) and *Pseudendoclonium akinetum* (Ulotrichales; according to [68]) formed a clade in all analyses. The topology of the tree was in general agreement with previous chloroplast phylogenomic analyses [3],[4],[53] in recovering the Pedinophyceae and the Chlorellales as early branching lineages of the core Chlorophyta, and the Chlorophyceae and core Trebouxiophyceae as strongly supported clades within the core Chlorophyta. Similar to the analysis of Fučíková et al. [3], but in contrast to the analysis of Cocquyt et al. [5], the Ulvophyceae fell apart into several clades, including the Bryopsidales (*Bryopsis*, *Codium*, *Halimeda*), Dasycladales (*Acetabularia*), Trentepohliales (*Cephaleuros*, *Trentepohlia*), and Ulvales (*Ulva*, *Pseudendoclonium*). The relationship among these clades was not well supported. A notable difference between our tree and the phylogeny of Fučíková et al. [3] was the position of *Oltmannsiellopsis* (Oltmannsiellopsidales) and *Tetraselmis*. *Oltmannsiellopsis* is currently classified as an ulvophyte, which was supported by chloroplast phylogenomic analyses (e.g. [4];[23]). *Tetraselmis* is a member of the Chlorodendrophyceae and in nuclear ribosomal DNA based phylogenies has been recovered as an early branching clade of the core Chlorophyta [2], [69]. The analyses of Fučíková et al. [3] unexpectedly supported a clade including *Tetraselmis* and *Oltmannsiellopsis*, which was found branching early in the radiation of the core Chlorophyta. In contrast, our analysis places *Oltmannsiellopsis* and *Tetraselmis* in the vicinity of the Ulvales-Ulotrichales. Our nucleotide based phylogenetic analysis placed *Tetraselmis* sister to the *Oltmannsiellopsis*-Ulvales-Ulotrichales clade (Fig 3), where as the AA based phylogeny recovered a *Tetraselmis*-*Oltmannsiellopsis* clade that was sister to the Ulvales-Ulotrichales clade. It should be noted that taxon sampling in the Ulvophyceae is still poor compared to the Trebouxiophyceae and Chlorophyceae, and that gene sampling in *Tetraselmis* is incomplete. Future studies with additional gene and taxon sampling will undoubtedly shed light on the relationships among the Ulvophyceae and the phylogenetic positions of the Oltmannsiellopsidales and Chlorodendrophyceae.

## Conclusion

Organellar genomes contain valuable information that can be used for understanding the evolution of these organelles as well as phylogenomic inferences of the green algal lineages. Our investigation of the chloroplast and mitochondrial genomes of *Ulva* sp. has added to the limited amount of genomic data available for the Ulvophyceae. We hope that these genomes will be useful for exploring primer sets for future molecular investigations to aid in species delimitation of *Ulva*, especially since only limited mitochondrial data is currently available for these taxonomically troublesome algae. As more genomes are published in the near future, we will gain further insights into the evolution of the highly rearranged mitochondrial and chloroplast genomes of green algae, and hopefully answer the major phylogenetic questions that still remain in the green algae tree of life.

## Supporting Information

S1 Fig Herbarium voucher of *Ulva* sp. UNA00071828. (PDF)

S2 Fig Neighbor-joining tree based on the *rbc*L gene (1000 bootstrap replicates). (PDF)

S3 Fig Neighbor-joining tree based on the *tuf*A gene (1000 bootstrap replicates). (PDF)

S4 Fig Genetic distances of the *rbc*L sequences of *Ulva* spp., *Monostroma grevillei* (GU183089) and *Blidingia minima* (AF387109) as outgroups. (PDF)

S5 Fig Genetic distances of the *tuf*A sequences of *Ulva* spp., *Monostroma* sp. (HQ610262) and *Blidingia minima* (HQ610239) as outgroups. (PDF)

S6 Fig Mauve alignments of the *Ulva* sp. cpDNA (top of alignment) with other chlorophytes. (A) *Pseudendoclonium akinetum* (NC_008114), (B) *Oltmannsiellopsis viridis* (NC_008099), (C) *Bryopsis hypnoides* (NC_013359), (D) *Chlorella vulgaris* (NC_001865), (E) *Oedogonium cardiacum* (NC_011031), (F) *Acutodesmus obliquus* (NC_008101), (G) *Pedinomonas minor* (NC_025530), (H) *Pycnococcus provasolii* (NC_012097), and (I) *Ostreococcus tauri* (NC_008289).
(PDF)

S7 Fig Mauve alignments of *Ulva* sp. mtDNA (top of aligment) with other chlorophytes. (A) *Pseudendoclonium akinetum* (NC_005926), (B) *Oltmannsiellopsis viridis* (NC_008256), (C) *Chlorella sorokiniana* (NC_024626), (D) *Prototheca wickerhamii* (NC_001613), (E) *Helicosporidium* sp. (NC_017841), (F) Trebouxiophyceae sp. MX-AZ01 (NC_018568), (G) *Pedinomonas minor* (NC_000892), (H) *Acutodesmus obliquus* (NC_002254), (I) *Ostreococcus tauri* (NC_008290), (J) *Nephroselmis olivacea* (NC_008239), (K) *Micomonas* sp. RCC299 (NC_012643), and (L) *Pycnococcus provasolii* (NC_013935).
(PDF)

S8 Fig ML tree with bootstrap values based on AA alignment of 51 chlorophyte genes. (PDF)

S9 Fig Bayesian tree with posterior probabilities based on AA alignment of 51 chlorophyte genes. (PDF)

S1 Table GenBank accession numbers used in the *rbc*L and *tuf*A phylogenetic trees. (PDF)

S2 Table *Ulva* sp. chloroplast tRNAs compared with *Oltmannsiellopsis viridis* and *Pseudendoclonium akinetum* tRNAs. (PDF)

S3 Table *Ulva* sp. mitochondrial tRNAs compared with *Oltmannsiellopsis viridis* and *Pseudendoclonium akinetum* tRNAs. (PDF)

S4 Table DCJ values calculated by UniMoG of chlorophyte cpDNAs without tRNAs. (PDF)

S5 Table DCJ values calculated by UniMoG of chlorophyte mtDNAs without tRNAs. (PDF)

S6 Table GenBank accession numbers of the taxon sampling for phylogenomics. (PDF)

## References

[1] Lewis LA, McCourt RM (2004) Green algae and the origin of land plants. Am J Bot 91: 1535–1556. 10.3732/ajb.91.10.1535 21652308

[2] Leliaert F, Smith DR, Moreau H, Herron MD, Verbruggen H, Delwiche CF, et al (2012) Phylogeny and Molecular Evolution of the Green Algae. Crit Rev Plant Sci 31: 1–46.

[3] Fučíková K, Leliaert F, Cooper ED, Škaloud P, D'hondt S, De Clerck O, et al (2014) New phylogenetic hypotheses for the core Chlorophyta based on chloroplast sequence data. Front Ecol Evol 2: 63.

[4] Lemieux C, Otis C, Turmel M (2014) Chloroplast phylogenomic analysis resolves deep-level relationships within the green algal class Trebouxiophyceae. BMC Evol Biol 14: 211 10.1186/s12862-014-0211-2 25270575 PMC4189289

[5] Cocquyt E, Verbruggen H, Leliaert F, De Clerck O (2010) Evolution and cytological diversification of the green seaweeds (Ulvophyceae). Mol Biol Evol 27: 2052–2061. 10.1093/molbev/msq091 20368268

[6] Ruhfel BR, Gitzendanner MA, Soltis PS, Soltis DE, Burleigh JG (2014) From algae to angiosperms-inferring the phylogeny of green plants (Viridiplantae) from 360 plastid genomes. BMC Evol Biol 14: 23 10.1186/1471-2148-14-23 24533922 PMC3933183

[7] Lang BF, Nedelcu AM (2012) Plastid Genomes of Algae In: Bock R, Knoop V, editors. Advances in Photosynthesis and Respiration Including Bioenergy and Related Processes: Genomics of Chloroplasts and Mitochondria. pp. 59–87. 10.1007/s11120-013-9921-3

[8] Burger G, Nedelcu AM (2012) Mitochondrial Genomes of Algae In: Bock R, Knoop V, editors. Advances in Photosynthesis and Respiration Including Bioenergy and Related Processes: Genomics of Chloroplasts and Mitochondria. pp. 125–157. 10.1007/s11120-013-9921-3

[9] Smith DR, Lee RW (2008) Mitochondrial genome of the colorless green alga *Polytomella capuana*: a linear molecule with an unprecedented GC content. Mol Biol Evol 25: 487–496. 10.1093/molbev/msm245 18222946

[10] Turmel M, Otis C, Lemieux C (2007) An unexpectedly large and loosely packed mitochondrial genome in the charophycean green alga *Chlorokybus atmophyticus* . BMC Genomics 12: 1–12. 10.1186/1471-2164-8-137 PMC1894977 17537252

[11] de Koning AP, Keeling PJ (2006) The complete plastid genome sequence of the parasitic green alga *Helicosporidium* sp. is highly reduced and structured. BMC Biology 4: 12 16630350 10.1186/1741-7007-4-12 PMC1463013

[12] Brouard J-S, Otis C, Lemieux C, Turmel M (2010) The exceptionally large chloroplast genome of the green alga *Floydiella terrestris* illuminates the evolutionary history of the Chlorophyceae. Genome Biol Evol 2: 240–256. 10.1093/gbe/evq014 20624729 PMC2997540

[13] Smith DR, Lee RW (2010) The mitochondrial and plastid genomes of *Volvox carteri*: bloated molecules rich in repetitive DNA. BMC Genomics 10: 132 10.1186/1471-2229-10-132 19323823 PMC2670323

[14] Padmanabhan U, Green BR (1978) The kinetic complexity of *Acetabularia* chloroplast DNA. BBA—Nucleic Acids and Protein Synthesis 521: 67–73. 10.1016/0005-2787(78)90249-6 363162

[15] Manhart JR, Kelly K, Dudock BS, Palmer JD (1989) Unusual characteristics of *Codium fragile* chloroplast DNA revealed by physical and gene mapping. Mol Gen Genet 216: 417–421. 2747622 10.1007/BF00334385

[16] Pombert J-F, Otis C, Lemieux C, Turmel M (2004) The complete mitochondrial DNA sequence of the green alga *Pseudendoclonium akinetum* (Ulvophyceae) highlights distinctive evolutionary trends in the Chlorophyta and suggests a sister-group relationship between the Ulvophyceae and Chlorophyceae. Mol Biol Evol 21: 922–935. 15014170 10.1093/molbev/msh099

[17] Pombert J-F, Beauchamp P, Otis C, Lemieux C, Turmel M (2006) The complete mitochondrial DNA sequence of the green alga *Oltmannsiellopsis viridis*: evolutionary trends of the mitochondrial genome in the Ulvophyceae. Curr Genet 50: 137–147. 16721603 10.1007/s00294-006-0076-z

[18] Guiry MD, Guiry GM (2014) AlgaeBase World-wide electronic publication, National University of Ireland, Galway Available: http://www.algaebase.org. Accessed 15 September 2014.

[19] Pombert J-F, Lemieux C, Turmel M (2006) The complete chloroplast DNA sequence of the green alga *Oltmannsiellopsis viridis* reveals a distinctive quadripartite architecture in the chloroplast genome of early diverging ulvophytes. BMC Biology 4: 3 16472375 10.1186/1741-7007-4-3 PMC1402334

[20] Pombert J-F, Otis C, Lemieux C, Turmel M (2005) The chloroplast genome sequence of the green alga *Pseudendoclonium akinetum* (Ulvophyceae) reveals unusual structural features and new insights into the branching order of chlorophyte lineages. Mol Biol Evol 22: 1903–1918. 15930151 10.1093/molbev/msi182

[21] Lü F, Xü W, Tian C, Wang G, Niu J, Pan G, et al (2011) The *Bryopsis hypnoides* plastid genome: multimeric forms and complete nucleotide sequence. PLoS ONE 6: e14663 10.1371/journal.pone.0014663 21339817 PMC3038852

[22] Lehman RL, Manhart JR (1997) A Preliminary Comparison of Restriction Fragment Patterns in the Genus *Caulerpa* (Chlorophyta) and the Unique Structure of the Chloroplast Genome of *Caulerpa sertulariodes* . J Phycol 33: 1055–1062. 9376187

[23] Zuccarello GC, Price N, Verbruggen H, Leliaert F (2009) Analysis of a plastid multigene data set and the phylogenetic position of the marine macroalga *Caulerpa filiformis* (Chlorophyta). J Phycol 45: 1206–1212. 27032364 10.1111/j.1529-8817.2009.00731.x

[24] Leliaert F, Zhang X, Ye N, Malta E-J, Engelen AH, Mineur F, et al (2009) Identity of the Qingdao algal bloom. Phycol Res 57: 147–151.

[25] Charlier RH, Morand P, Finkl CW, Thys A (2007) Green Tides on the Brittany Coasts. EREM 3: 52–59.

[26] Norris JN (2010) Marine Algae of the Northern Gulf of California: Chlorophyta and Phaeophyceae. Washington, D.C.: Smithsonian Institution Scholarly Press p. 31.

[27] Hayden H, Blomster J, Maggs CA, Silva PC, Stanhope MJ, Waaland JR (2003) Linnaeus was right all along: *Ulva* and *Enteromorpha* are not distinct genera. Eur J Phycol 38: 27–294. 12593914

[28] Hofmann L, Nettleton J, Neefus C, Mathieson A (2010) Cryptic diversity of *Ulva* (Ulvales, Chlorophyta) in the Great Bay Estuarine System (Atlantic USA): introduced and indigenous distromatic species. Eur J Phycol 45: 230–239.

[29] Heesch S, Broom JES, Neill KF, Farr TJ, Dalen JL, Nelson WA (2009) *Ulva*, *Umbraulva* and *Gemina*: genetic survey of New Zealand taxa reveals diversity and introduced species. Eur J Phycol 44: 143–154.

[30] Kraft LGK, Kraft GT, Waller RF (2010) Investigations Into Southern Australian *Ulva* (Ulvophyceae, Chlorophyta) Taxonomy and Molecular Phylogeny Indicate Both Cosmopolitanism and Endemic Cryptic Species. J Phycol 46: 1257–1277.

[31] O’Kelly CJ, Kurihara A, Shipley TC, Sherwood AR (2010) Molecular assessment of *Ulva* spp. (Ulvophyceae, Chlorophyta) in the Hawaiian Islands. J Phycol 46: 728–735.

[32] Saunders GW, Kucera H (2010) An evaluation of *rbc*L, *tuf*A, UPA, LSU and ITS as DNA barcode markers for the marine green macroalgae. Cryptogam, Algol 31: 487–528.

[33] Kirkendale L, Saunders GW, Winberg P (2012) A molecular survey of *Ulva* (Chlorophyta) in temperate Australia reveals enhanced levels of cosmopolitanism. J Phycol 49: 69–81. 27008390 10.1111/jpy.12016

[34] Guidone M, Thornber C, Wysor B, O’Kelly CJ (2013) Molecular and morphological diversity of Narragansett Bay (RI, USA) *Ulva* (Ulvales, Chlorophyta) populations. J Phycol 49: 979–995. 27007320 10.1111/jpy.12108

[35] Xu J, Fan X, Zhang X, Xu D, Mou S, Cao S, et al (2012) Evidence of coexistence of C_3_ and C_4_ photosynthetic pathways in a green-tide-forming alga, *Ulva prolifera* . PLoS ONE 7: e37438 10.1371/journal.pone.0037438 22616009 PMC3353924

[36] Zhang X, Ye N, Liang C, Mou S, Fan X, et al (2012) De novo sequencing and analysis of the *Ulva linza* transcriptome to discover putative mechanisms associated with its successful colonization of coastal ecosystems. BMC Genomics 13: 565 10.1186/1471-2164-13-565 23098051 PMC3532339

[37] Edgar RC (2004) MUSCLE: multiple sequence alignment with high accuracy and high throughput. Nucleic Acids Res 32: 1792–1797. 15034147 10.1093/nar/gkh340 PMC390337

[38] Kearse M, Moir R, Wilson A, Stones-Havas S, Cheung M, Sturrock S, et al (2012) Geneious Basic: an integrated and extendable desktop software platform for the organization and analysis of sequence data. Bioinformatics 28: 1647–1649. 10.1093/bioinformatics/bts199 22543367 PMC3371832

[39] Tamura K, Stecher G, Peterson D, Fiüipski A, Kumar S (2013) MEGA6: Molecular Evolutionary Genetics Analysis Version 6.0. Mol Biol Evol 30: 2725–2729. 10.1093/molbev/mst197 24132122 PMC3840312

[40] Lawton RJ, Mata L, de Nys R, Paul NA (2013) Algal bioremediation of waste waters from land-based aquaculture using *Ulva*: selecting target species and strains. PLoS ONE 8: e77344 10.1371/journal.pone.0077344 24143221 PMC3797103

[41] Trim Galore! v0.3.7. created by Babraham Bioinformatics. Available: http://www.bioinformatics.babraham.ac.uk/.

[42] Zerbino DR, Birney E (2008) Velvet: Algorithms for de novo short read assembly using de Bruijn graphs. Genome Res. 18: 821–829. 10.1101/gr.074492.107 18349386 PMC2336801

[43] CLC Genomics Workbench 7.0.3. Available: http://www.clcbio.com.

[44] Lowe TM, Eddy SR (1997) The tRNAscan-SE, snoscan and snoGPS web servers for the detection of tRNAs and snoRNAs. Nucleic Acids Res 25: 955–964. 15980563 10.1093/nar/gki366 PMC1160127

[45] Schattner P, Brooks AN, Lowe TM (2005) tRNAscan-SE: a Program for Improved Detection of Transfer RNA Genes in Genomic Sequence. Nucleic Acids Res 33: 686–689. 10.1093/nar/25.5.955 PMC146525 9023104

[46] Rice P, Longden I, Bleasby A (2000) EMBOSS: The European Molecular Biology Open Software Suite. Trends Genet 16: 276–277. 10827456 10.1016/s0168-9525(00)02024-2

[47] Lohse M, Drechsel O, Kahlau S, Bock R (2013) OrganellarGenomeDRAW–a suite of tools for generating physical maps of plastid and mitochondrial genomes and visualizing expression data sets. Nucleic Acids Research 10.1093/nar/gkt289 PMC3692101 23609545

[48] Braga MDV, Willing E, Stoye J (2011) Double cut and join with insertions and deletions. J Comput Biol 18: 1167–1184. 10.1089/cmb.2011.0118 21899423

[49] Hilker R, Sickinger C, Pedersen CNS, Stoye J (2012) UniMoG–a unifying framework for genomic distance calculation and sorting based on DCJ. Bioinformatics. 28: 2509–2511. 22815356 10.1093/bioinformatics/bts440 PMC3463123

[50] Bergeron A, Mixtacki J, Stoye J. A unifying view of genome rearrangements In: Bücher P, Moret BE, editors. Algorithms in Bioinformatics. Springer Berlin Heidelberg; 2006 pp. 163−173.

[51] Darling AE, Mau B, Blatter FR, Perna NT (2004) Mauve: multiple alignment of conserved genomic sequence with rearrangements. Genome Res 14: 1394–1403. 15231754 10.1101/gr.2289704 PMC442156

[52] Darling AE, Mau B, Perna NT (2010) progressiveMauve: multiple genome alignment with gene gain, loss, and rearrangement. PLoS ONE 5: e11147 10.1371/journal.pone.0011147 20593022 PMC2892488

[53] Lemieux C, Otis C, Turmel M (2014) Six newly sequenced chloroplast genomes from prasinophyte green algae provide insights into the relationships among prasinophyte lineages and the diversity of streamlined genome architecture in picoplanktonic species. BMC Genomics 15: 857 10.1186/1471-2164-15-857 25281016 PMC4194372

[54] Smith DR, Burki F, Yamada T, Grimwood J, Grigoriev IV, Van Etten JL, et al (2011) The GC-rich mitochondrial and plastid genomes of the green alga *Coccomyxa* give insight into the evolution of organelle DNA nucleotide landscape. PLoS ONE 6: e23624 10.1371/journal.pone.0023624 21887287 PMC3162594

[55] Larkin MA, Blackshields G, Brown NP, Chenna R, McGettigan PA, McWilliam H, et al (2007) Clustal W and Clustal X version 2.0. Bioinformatics 23: 2947–2948. 17846036 10.1093/bioinformatics/btm404

[56] Castresana J (2000) Selection of conserved blocks from multiple alignments for their use in phylogenetic analysis. Mol Biol Evol 17: 540–552. 10742046 10.1093/oxfordjournals.molbev.a026334

[57] Stamatakis A (2006) RAxML-VI-HPC: Maximum likelihood-based phylogenetic analyses with thousands of taxa and mixed models. Bioinformatics 22: 2688–2690. 16928733 10.1093/bioinformatics/btl446

[58] Ronquist F, Huelsenbeck JP (2003) MrBayes 3: Bayesian phylogenetic inference under mixed models. Bioinformatics 19: 1572–1574. 12912839 10.1093/bioinformatics/btg180

[59] Rambaut A, Drummond AJ (2007) Tracer v1.4, Available from http://beast.bio.ed.ac.uk/Tracer.

[60] Servín-Garcidueñas LE, Martínez-Romero E (2012) Complete Mitochondrial and Plastid Genomes of the Green Microalga Trebouxiophyceae sp. Strain MX-AZ01 Isolated from a Highly Acidic Geothermal Lake. Eukaryot Cell 11: 1417–1418. 10.1128/EC.00244-12 23104370 PMC3486031

[61] Wolff G, Plante I, Lang BF, Kück U, Burger G (1994) Complete sequence of the mitochondrial DNA of the Chlorophyte Alga *Prototheca wickerhamii*: gene content and genome organization. J Mol Biol 237: 75–86. 8133522 10.1006/jmbi.1994.1210

[62] Pombert J-F, Keeling PJ (2010) The Mitochondrial Genome of the Entomoparasitic Green Alga *Helicosporidium* . PLoS ONE. 10.1371/journal.pone.0008954 PMC2813288 20126458

[63] Liu Y, Xue J, Wang B, Li L, Qiu Y-L (2012) Conservative and Dynamic Evolution of Mitochondrial Genomes in Early Land Plants In: Bock R, Knoop V, editors. Advances in Photosynthesis and Respiration Including Bioenergy and Related Processes: Genomics of Chloroplasts and Mitochondria. pp. 159–174. 10.1007/s11120-013-9921-3

[64] Knoop V. Seed Plant Mitochondrial Genomes: Complexity Evolving In: Bock R, Knoop V, editors. Advances in Photosynthesis and Respiration Including Bioenergy and Related Processes: Genomics of Chloroplasts and Mitochondria. Springer Dordrecht Heidelberg New York London; 2012 pp. 175–200. 10.1007/s11120-013-9921-3

[65] Alverson AJ, Rice DW, Dickinson S, Barry K, Palmer JD (2011) Origins and recombination of the bacterial-sized multichromosomal mitochondrial genome of cucumber. Plant Cell 23: 2499–2513. 10.1105/tpc.111.087189 21742987 PMC3226218

[66] DePriest MS, Bhattacharya D, Lopez-Bautista JM (2013) The Plastid Genome of the Red Macroalga *Grateloupia taiwanensis* (Halymeniaceae). PLoS ONE 8: e68246 10.1371/journal.pone.0068246 23894297 PMC3716797

[67] DePriest MS, Bhattacharya D, Lopez-Bautista JM (2014) The Mitochondrial Genome of *Grateloupia taiwanensis* (Halymeniaceae, Rhodophyta) and Comparative Mitochondrial Genomic of Red Algae. Biol Bull 227: 191–200. 25411376 10.1086/BBLv227n2p191

[68] Škaloud P, Nedbalová L, Elster J, Komárek J (2013) A curious occurrence of *Hazenia broadyi* spec. nova in Antarctica and the review of the genus *Hazenia* (Ulotrichales, Chlorophyceae). Polar Biol 36: 1281–1291.

[69] Marin B (2012) Nested in the Chlorellales or independent class? Phylogeny and classification of the Pedinophyceae (Viridiplantae) revealed by molecular phylogenetic analyses of complete nuclear and plastid-encoded rRNA operons. Protist 163: 778–805. 10.1016/j.protis.2011.11.004 22192529

