# Supplementary figures and images for: The Complete Chloroplast and Mitochondrial Genomes of the Green Macroalga Ulva sp. UNA00071828 (Ulvophyceae, Chlorophyta)
Source: PLoS One. 2015 Apr 7;10(4):e0121020. doi: 10.1371/journal.pone.0121020 (PMC4388391; doi:10.1371/journal.pone.0121020)

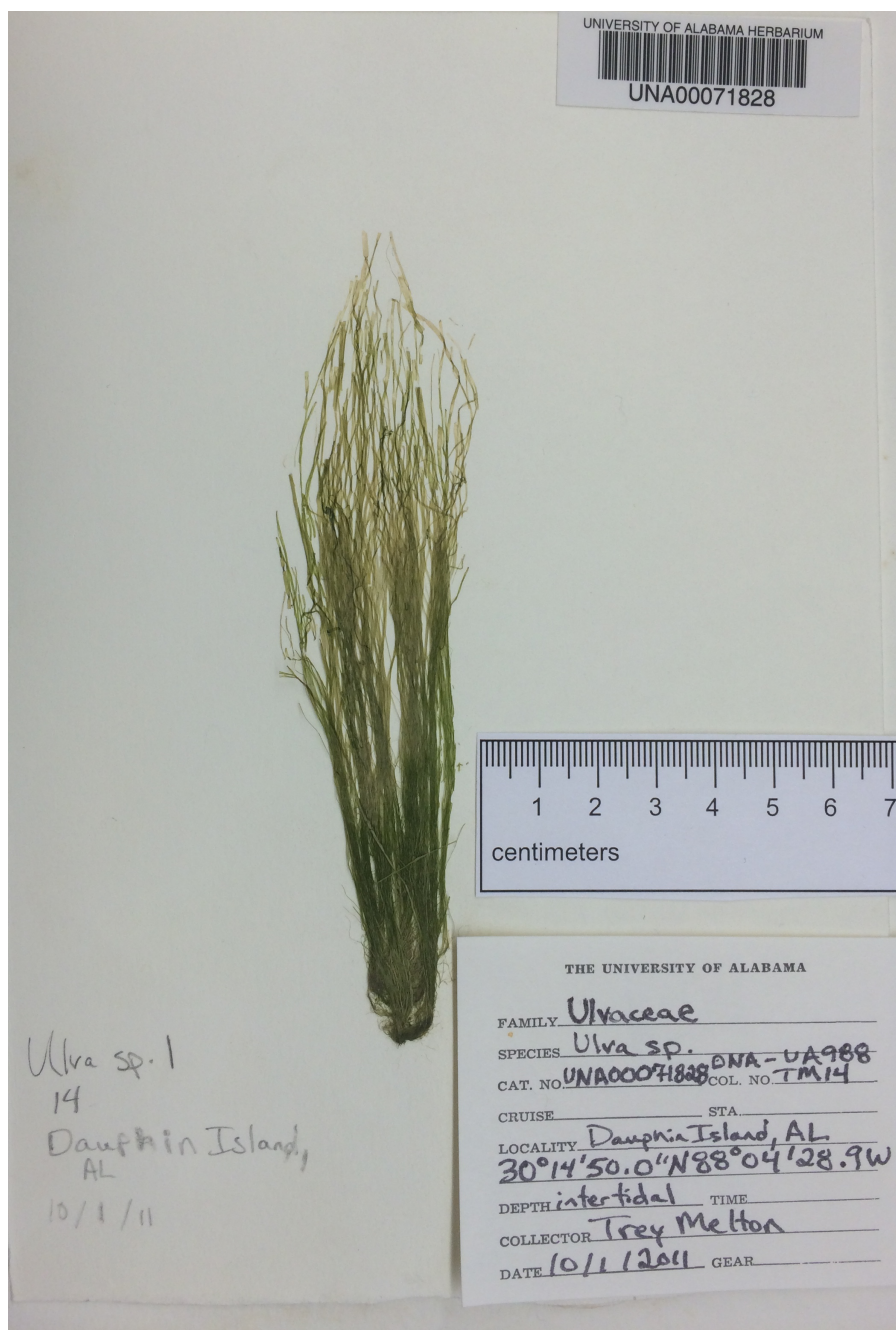

S1 Fig. Herbarium voucher of *Ulva* sp. UNA00071828.

Supplement: S1 Fig — (PDF) [file pone.0121020.s001.pdf]

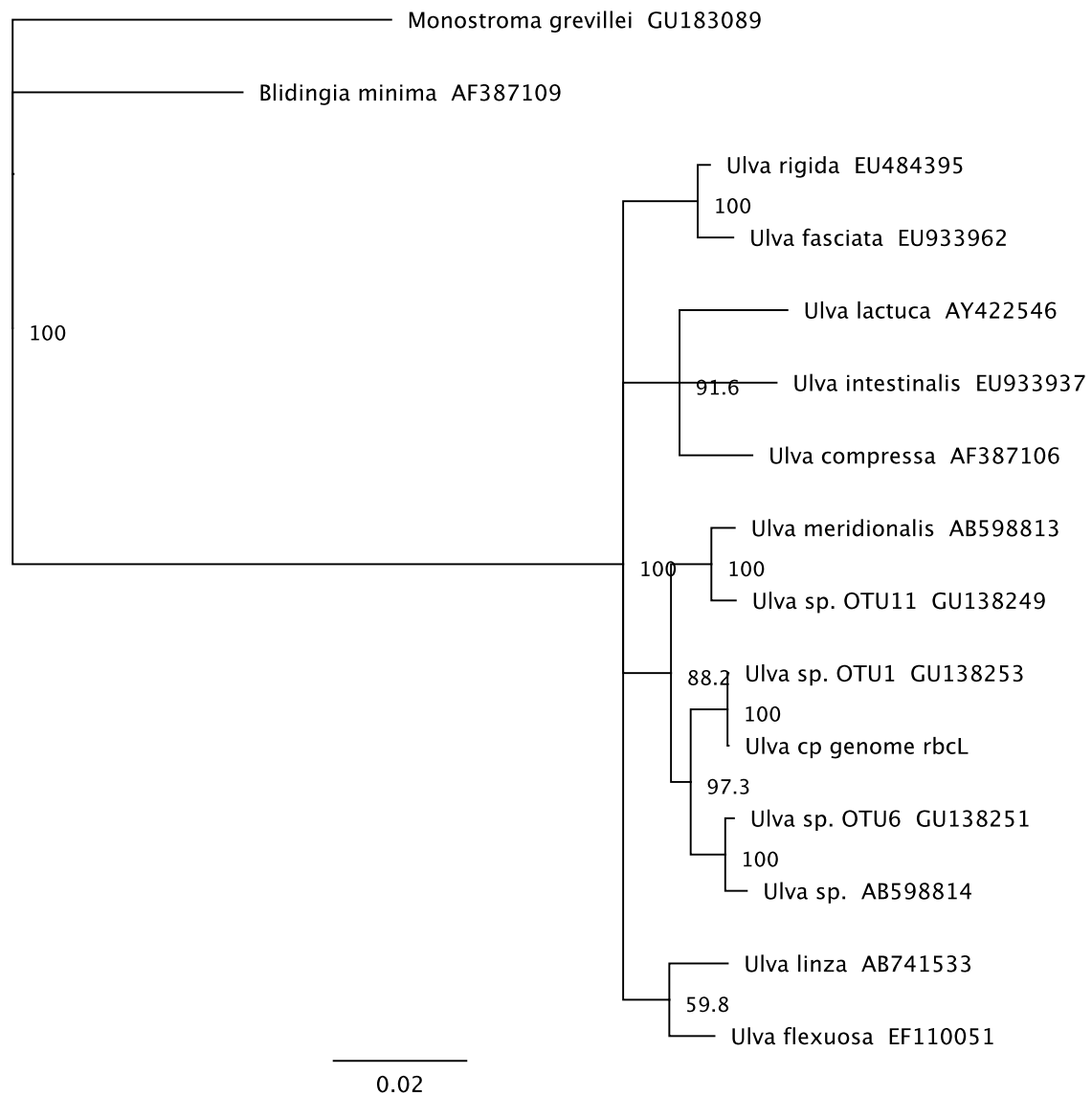

**S2 Fig. Neighbor-joining tree based on the *rbcL* gene (1000 bootstrap replicates).**

Supplement: S2 Fig — (PDF) [file pone.0121020.s002.pdf]

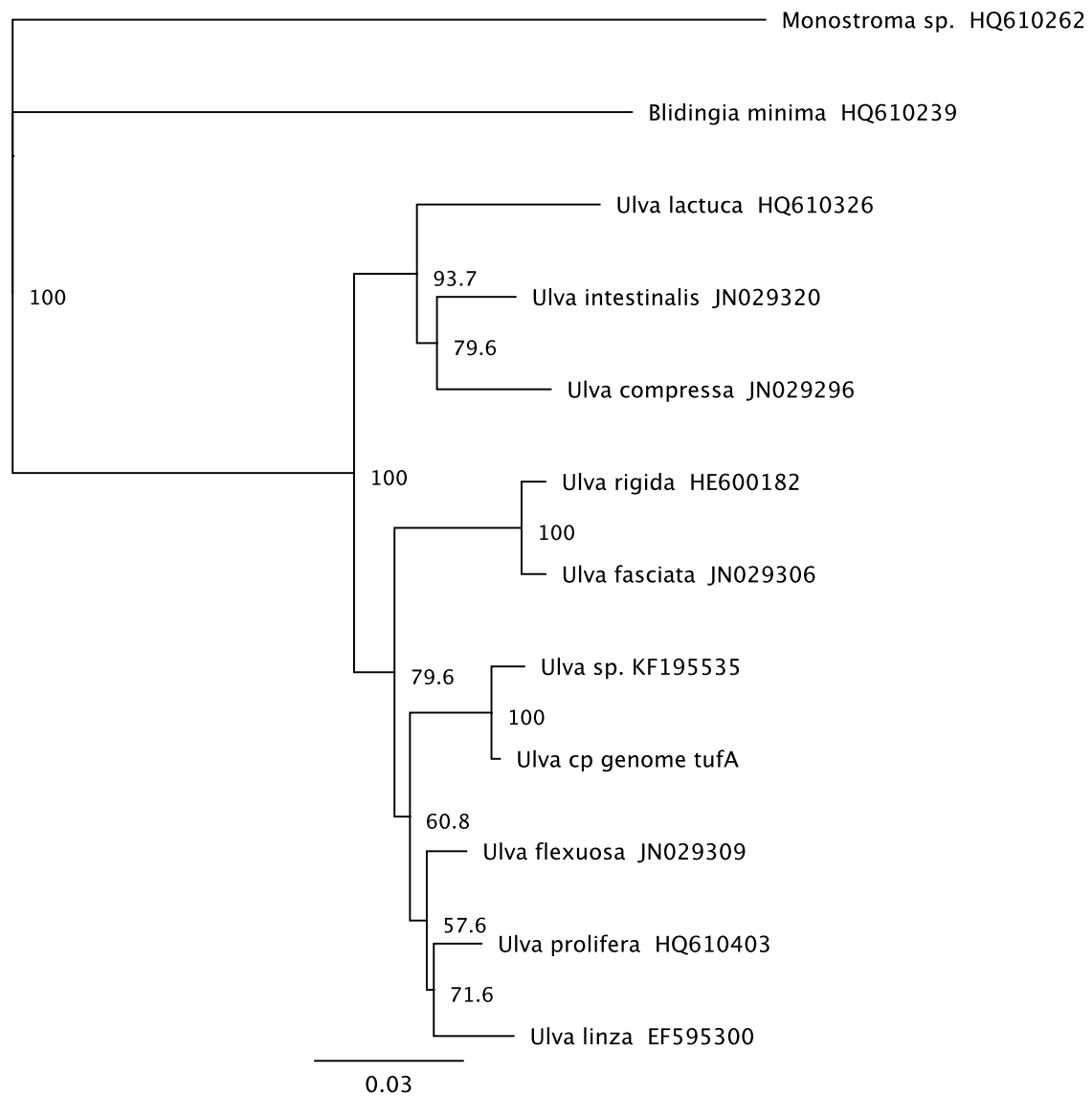

**S3 Fig. Neighbor-joining tree based on the *tufA* gene (1000 bootstrap replicates).**

Supplement: S3 Fig — (PDF) [file pone.0121020.s003.pdf]

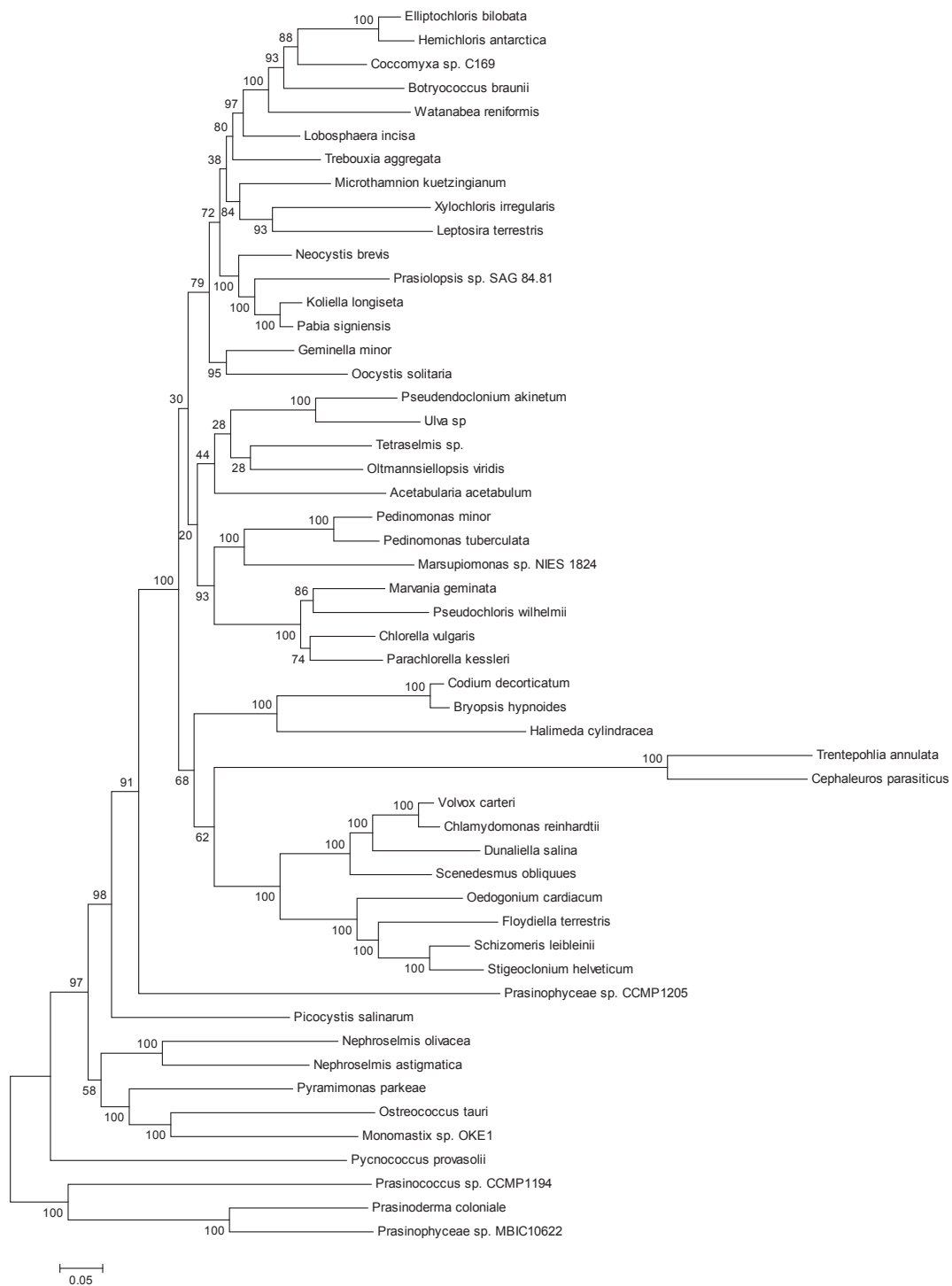

**S8 Fig. ML tree with bootstrap values based on AA alignment of 51 chlorophyte genes.**

Supplement: S8 Fig — (PDF) [file pone.0121020.s008.pdf]
