# Supplementary material for: The Complete Chloroplast and Mitochondrial Genomes of the Green Macroalga Ulva sp. UNA00071828 (Ulvophyceae, Chlorophyta)
Source: PLoS One. 2015 Apr 7;10(4):e0121020. doi: 10.1371/journal.pone.0121020 (PMC4388391; doi:10.1371/journal.pone.0121020)
Supplement: S4 Fig — (PDF) [file pone.0121020.s004.pdf]

M6: Pairwise Distances (C:\users\treymelton\Desktop\Dropbox\Ulva_genome_rbcL2.meg)

File Display Average Caption Help

	1	2	3	4	5	6	7	8	9	10	11	12	13	14
1. Ulva meridionalis AB598813														
2. Ulva flexuosa EF110051	27.000													
3. Ulva linza AB741533	31.000	22.000												
4. Ulva rigida EU484395	28.000	23.000	30.000											
5. Ulva fasciata EU933962	33.000	26.000	33.000	8.000										
6. Ulva sp. OTU1 GU138253	23.000	27.000	31.000	29.000	34.000									
7. Ulva sp. OTU6 GU138251	22.000	27.000	32.000	30.000	35.000	14.000								
8. Ulva sp. OTU11 GU138249	8.000	30.000	33.000	28.000	32.000	22.000	23.000							
9. Ulva sp. AB598814	25.000	28.000	33.000	31.000	36.000	15.000	5.000	26.000						
10. Ulva lactuca AY422546	39.000	34.000	37.000	34.000	38.000	37.000	39.000	38.000	39.000					
11. Monostroma grevillei GU183089	169.000	168.000	163.000	164.000	163.000	160.000	160.000	164.000	163.000	170.000				
12. Blidingia minima AF387109	144.000	145.000	143.000	138.000	140.000	143.000	145.000	144.000	147.000	151.000	92.000			
13. Ulva intestinalis EU933937	40.000	35.000	40.000	34.000	37.000	40.000	42.000	39.000	43.000	29.000	168.000	142.000		
14. Ulva compressa AF387106	32.000	25.000	31.000	34.000	39.000	36.000	36.000	35.000	37.000	29.000	168.000	143.000	28.000	
15. Ulva cp genome rbcL	23.000	27.000	31.000	29.000	34.000	0.000	14.000	22.000	15.000	37.000	160.000	143.000	40.000	36.000

S4 Fig. Genetic distances of the *rbc*L sequences of *Ulva* spp., *Monostroma grevillei* (GU183089) and *Blidingia minima* (AF387109) as outgroups.
