# Supplementary material for: The Complete Chloroplast and Mitochondrial Genomes of the Green Macroalga Ulva sp. UNA00071828 (Ulvophyceae, Chlorophyta)
Source: PLoS One. 2015 Apr 7;10(4):e0121020. doi: 10.1371/journal.pone.0121020 (PMC4388391; doi:10.1371/journal.pone.0121020)
Supplement: S5 Fig — (PDF) [file pone.0121020.s005.pdf]

M6: Pairwise Distances (C:\users\treymelton\Desktop\Dropbox\Ulva_genome_tufA2.meg)

	1	2	3	4	5	6	7	8	9	10	11
1. Ulva prolifera HQ610403											
2. Ulva linza EF595300	19.000										
3. Ulva flexuosa JN029309	15.000	19.000									
4. Ulva rigida HE600182	35.000	36.000	30.000								
5. Ulva fasciata JN029306	35.000	37.000	31.000	7.000							
6. Ulva lactuca HQ610326	51.000	53.000	51.000	60.000	57.000						
7. Ulva intestinalis JN029320	41.000	47.000	40.000	43.000	43.000	40.000					
8. Ulva compressa JN029296	46.000	53.000	44.000	45.000	46.000	44.000	28.000				
9. Monostroma sp. HQ610262	147.000	154.000	154.000	159.000	160.000	159.000	155.000	157.000			
10. Blidingia minima HQ610239	135.000	139.000	137.000	145.000	146.000	153.000	139.000	149.000	167.000		
11. Ulva sp. KF195535	30.000	35.000	23.000	41.000	40.000	57.000	45.000	50.000	155.000	140.000	
12. Ulva cp genome tufA	26.000	31.000	19.000	38.000	37.000	55.000	43.000	45.000	152.000	137.000	6.000

S5 Fig. Genetic distances of the *tuf*A sequences of *Ulva* spp., *Monostroma* sp. (HQ610262) and *Blidingia minima* (HQ610239) as outgroups.
