# Supplementary material for: The Complete Chloroplast and Mitochondrial Genomes of the Green Macroalga Ulva sp. UNA00071828 (Ulvophyceae, Chlorophyta)
Source: PLoS One. 2015 Apr 7;10(4):e0121020. doi: 10.1371/journal.pone.0121020 (PMC4388391; doi:10.1371/journal.pone.0121020)
Supplement: S6 Fig — (A) Pseudendoclonium akinetum (NC_008114), (B) Oltmannsiellopsis viridis (NC_008099), (C) Bryopsis hypnoides (NC_013359), (D) Chlorella vulgaris (NC_001865), (E) Oedogonium cardiacum (NC_011031), (F) Acutodesmus obliquus (NC_008101), (G) Pedinomonas minor (NC_025530), (H) Pycnococcus provasolii (NC_012097), and (I) Ostreococcus tauri (NC_008289). (PDF) [file pone.0121020.s006.pdf]

S6 Fig. Mauve alignments of the *Ulva* sp. cpDNA (top of alignment) with other chlorophytes. (A) *Pseudendoclonium akinetum* (NC_008114), (B) *Oltmannsiellopsis viridis* (NC_008099), (C) *Bryopsis hypnoides* (NC_013359), (D) *Chlorella vulgaris* (NC_001865), (E) *Oedogonium cardiacum* (NC_011031), (F) *Acutodesmus obliquus* (NC_008101), (G) *Pedinomonas minor* (NC_025530), (H) *Pycnococcus provasolii* (NC_012097), and (I) *Ostreococcus tauri* (NC_008289).

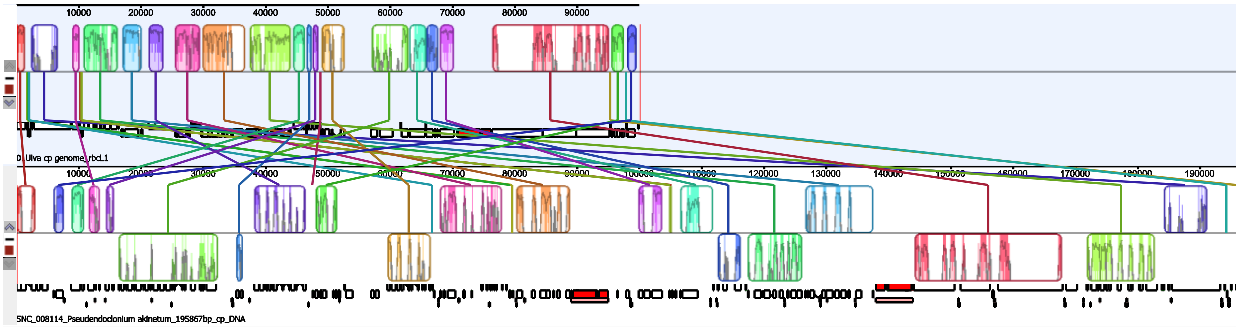

A)

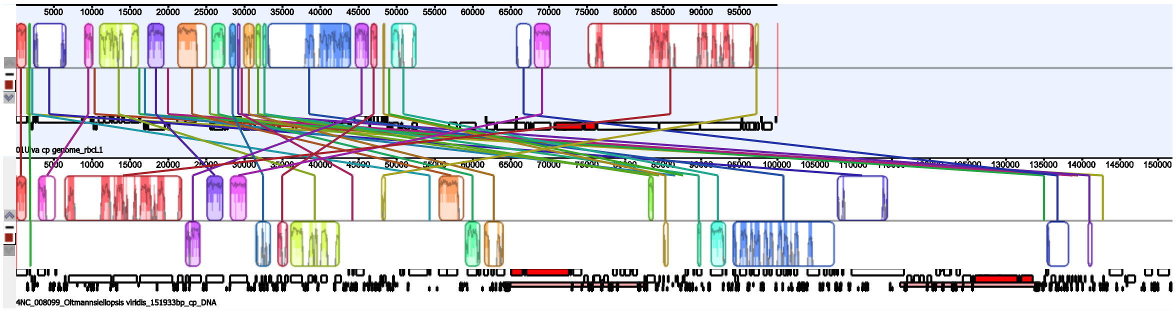

B)

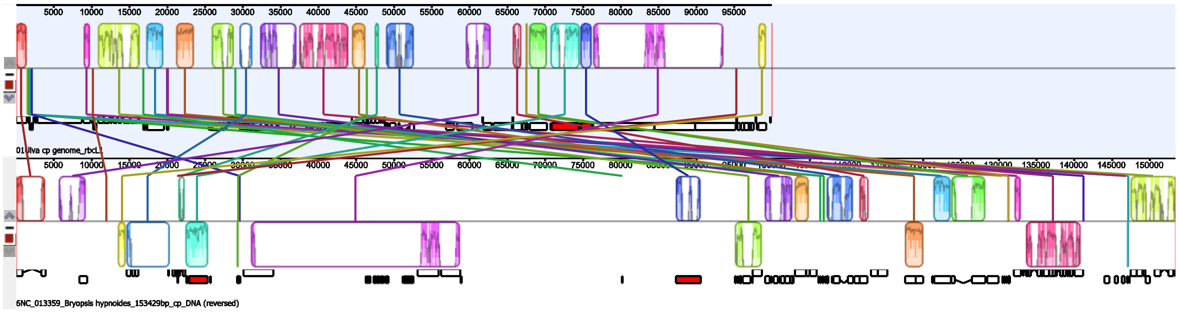

C)

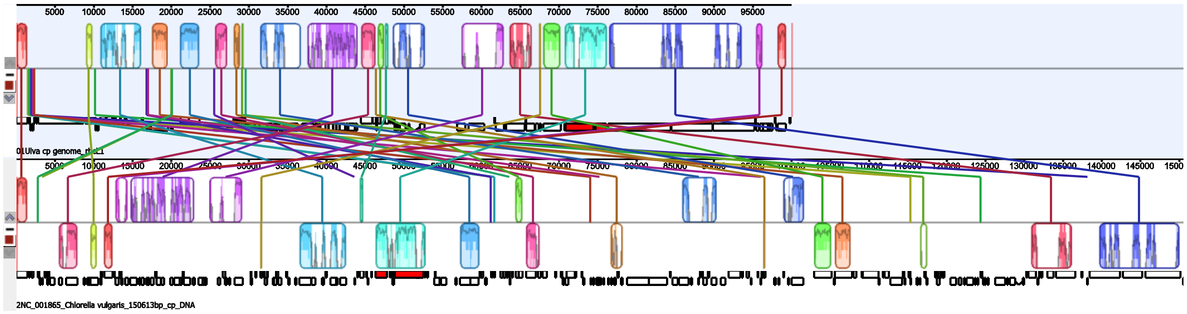

D)

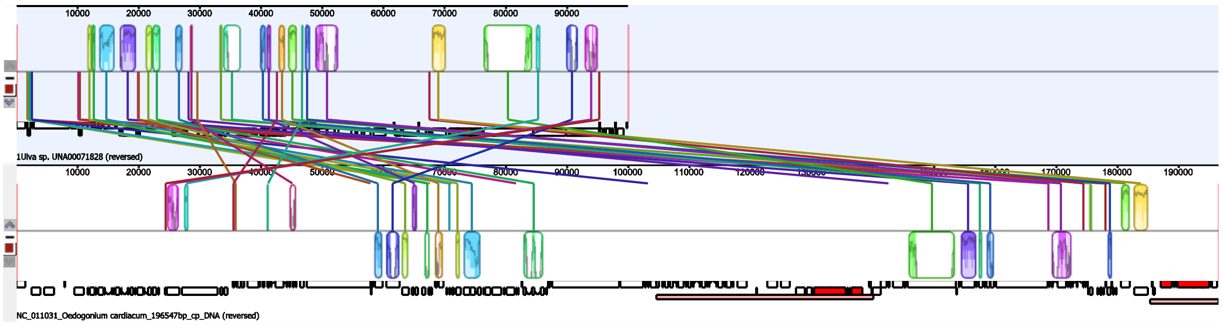
1Ulva sp. UNA00071828 (reversed)
NC_011031_Oedogonium cardiacum_196547bp_cp_DNA (reversed)

E)

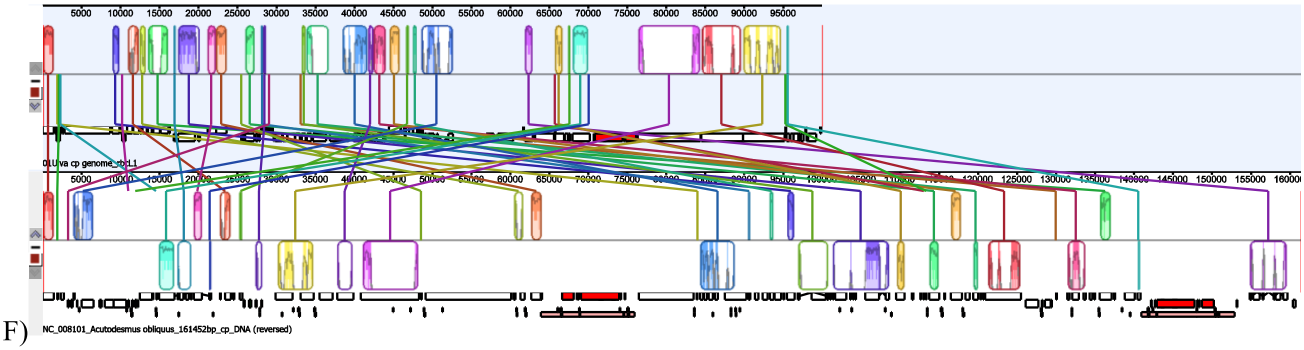
01Ulva cp genome_rbcL1
NC_008101_Acutodesmus obliquus_161452bp_cp_DNA (reversed)

F)

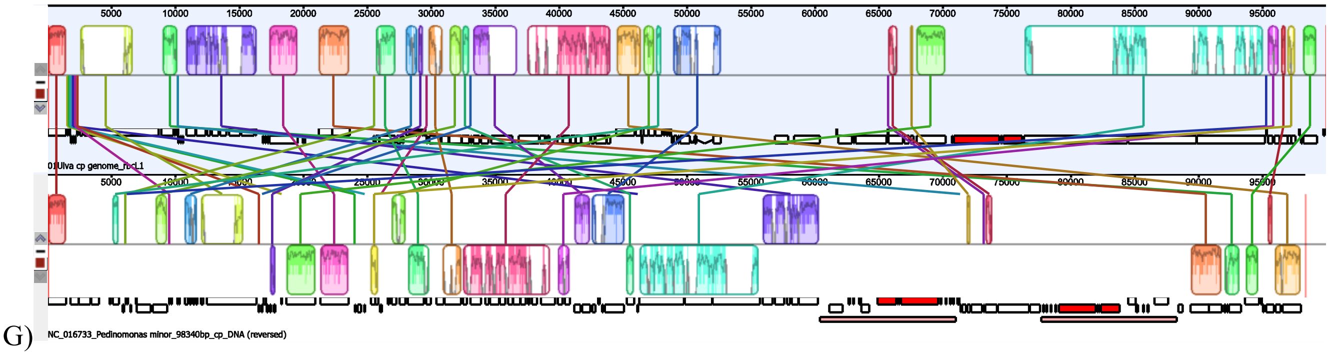
01Ulva cp genome_rbcL1
NC_016733_Pedinomonas minor_98340bp_cp_DNA (reversed)

G)

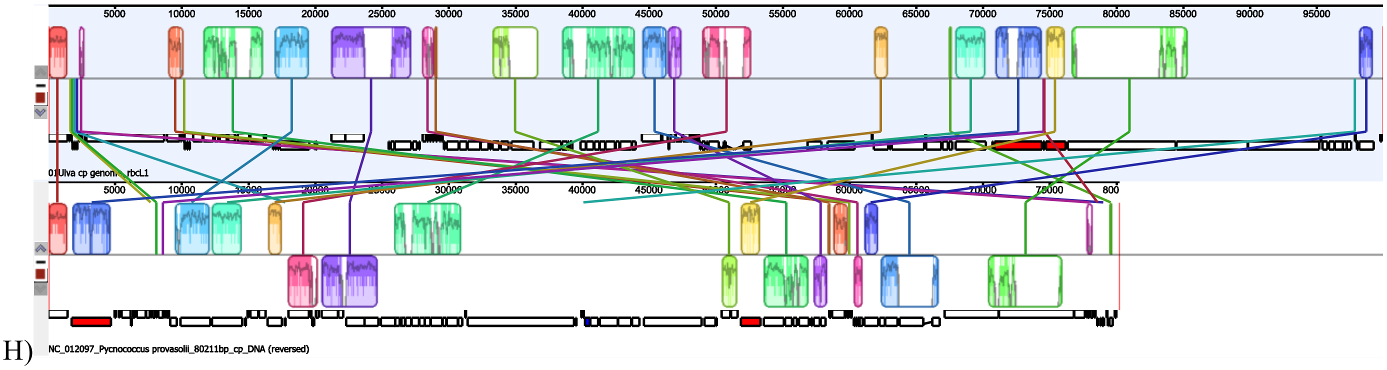
NC_012097_Pycnococcus provasolii_80211bp_cp_DNA (reversed)

H)

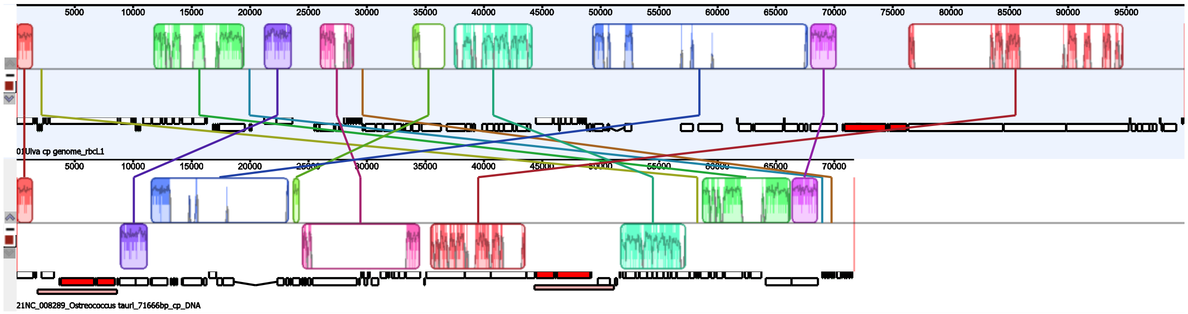
01Ulva cp genome_rbcL1
21NC_008289_Ostreococcus tauri_71666bp_cp_DNA

I)
