# Supplementary material for: The Complete Chloroplast and Mitochondrial Genomes of the Green Macroalga Ulva sp. UNA00071828 (Ulvophyceae, Chlorophyta)
Source: PLoS One. 2015 Apr 7;10(4):e0121020. doi: 10.1371/journal.pone.0121020 (PMC4388391; doi:10.1371/journal.pone.0121020)
Supplement: S7 Fig — (A) Pseudendoclonium akinetum (NC_005926), (B) Oltmannsiellopsis viridis (NC_008256), (C) Chlorella sorokiniana (NC_024626), (D) Prototheca wickerhamii (NC_001613), (E) Helicosporidium sp. (NC_017841), (F) Trebouxiophyceae sp. MX-AZ01 (NC_018568), (G) Pedinomonas minor (NC_000892), (H) Acutodesmus obliquus (NC_002254), (I) Ostreococcus tauri (NC_008290), (J) Nephroselmis olivacea (NC_008239), (K) Micomonas sp. RCC299 (NC_012643), and (L) Pycnococcus provasolii (NC_013935). (PDF) [file pone.0121020.s007.pdf]

S7 Fig. Mauve alignments of *Ulva* sp. mtDNA (top of aligment) with other chlorophytes. (A) *Pseudendoclonium akinetum* (NC_005926), (B) *Oltmannsiellopsis viridis* (NC_008256), (C) *Chlorella sorokiniana* (NC_024626), (D) *Prototheca wickerhamii* (NC_001613), (E) *Helicosporidium* sp. (NC_017841), (F) Trebouxiophyceae sp. MX-AZ01 (NC_018568), (G) *Pedinomonas minor* (NC_000892), (H) *Acutodesmus obliquus* (NC_002254), (I) *Ostreococcus tauri* (NC_008290), (J) *Nephroselmis olivacea* (NC_008239), (K) *Micomonas* sp. RCC299 (NC_012643), and (L) *Pycnococcus provasolii* (NC_013935).

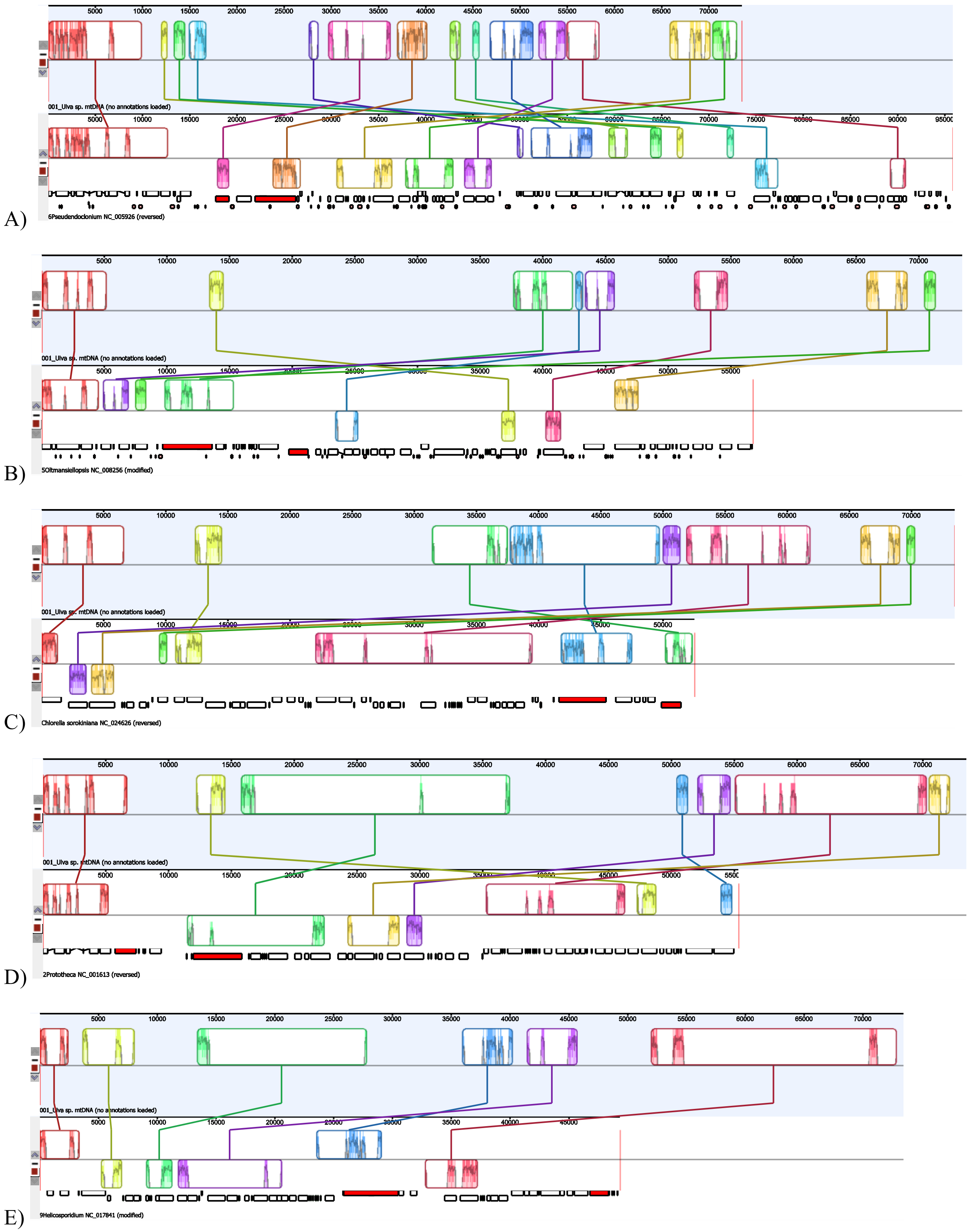

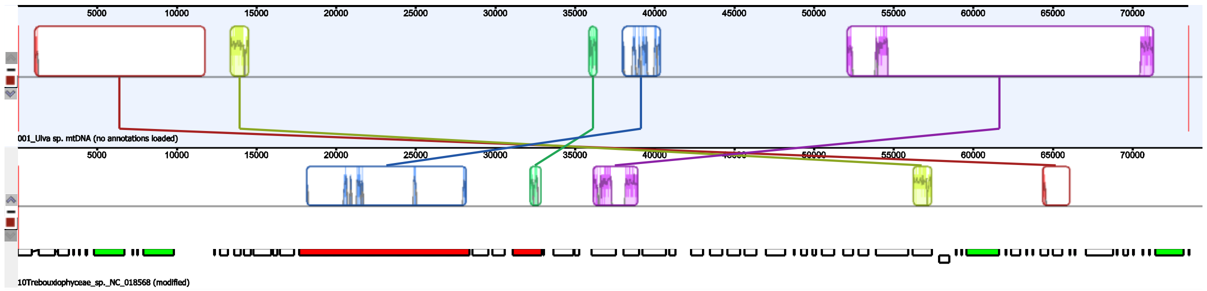
001_Ulva sp. mtDNA (no annotations loaded)
10Trebouxiophyceae_sp._NC_018568 (modified)
F)

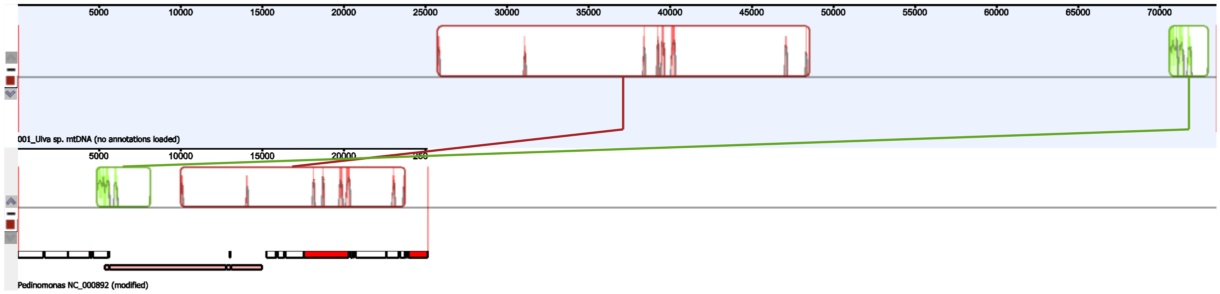
001_Ulva sp. mtDNA (no annotations loaded)
Pedinomonas NC_000892 (modified)
G)

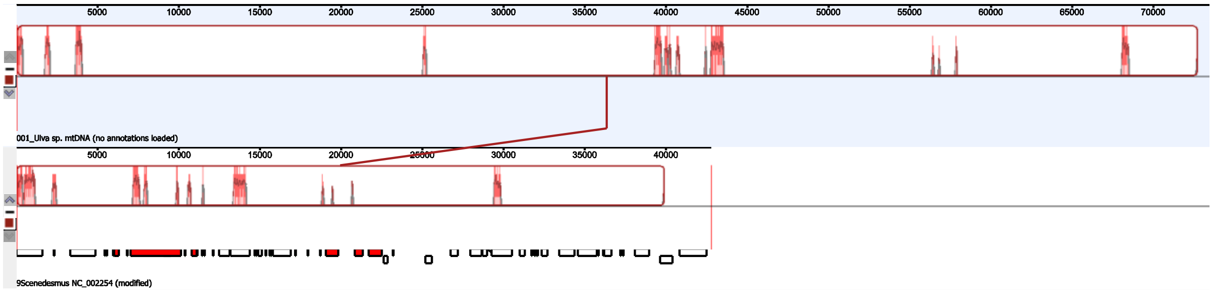
001_Ulva sp. mtDNA (no annotations loaded)
9Scenedesmus NC_002254 (modified)
H)

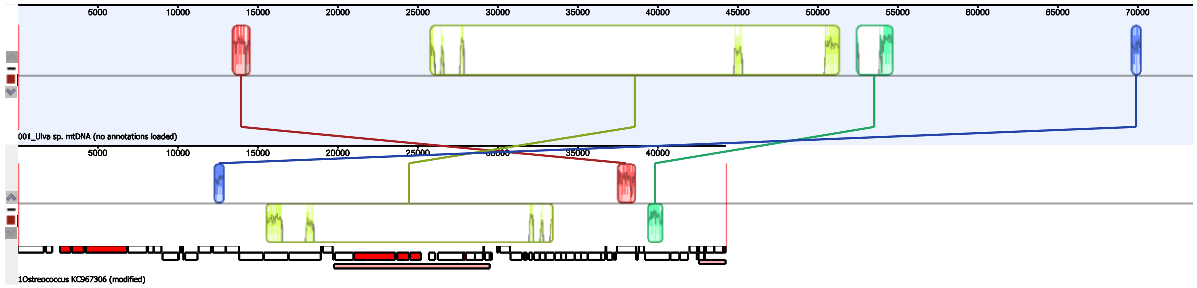
001_Ulva sp. mtDNA (no annotations loaded)
1Ostreococcus KC967306 (modified)
I)

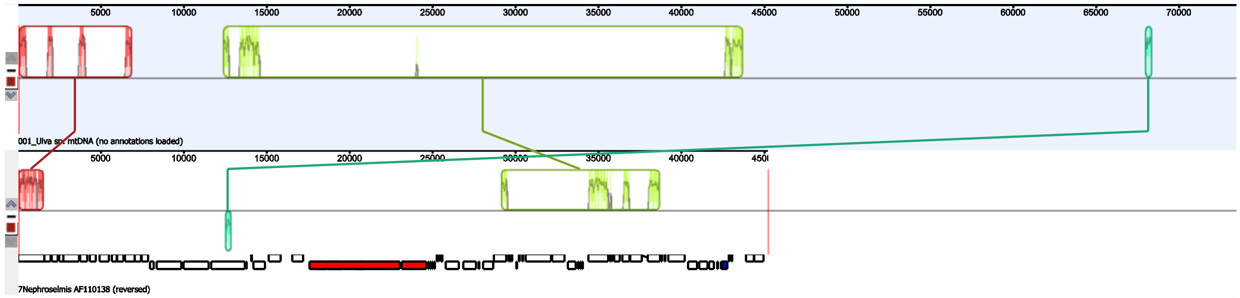
7Nephroselmis AF110138 (reversed)
J)

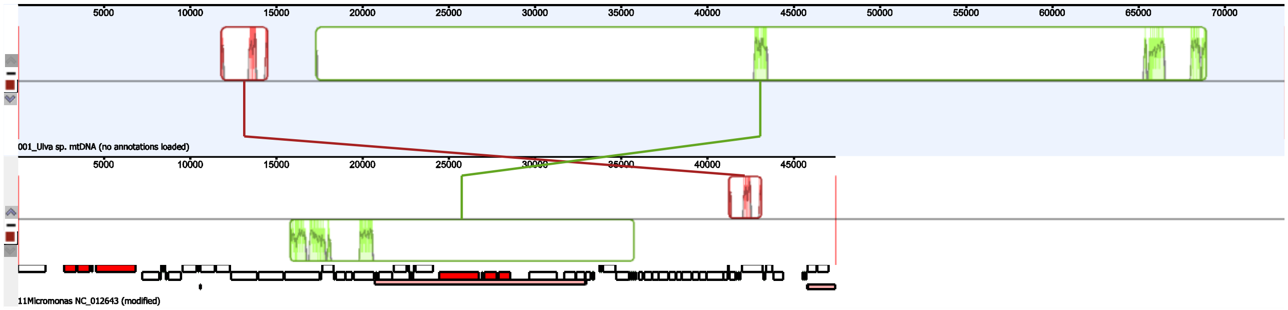
5000
10000
15000
20000
25000
30000
35000
40000
45000
50000
55000
60000
65000
70000
001_Ulva sp. mtDNA (no annotations loaded)
11Micromonas NC_012643 (modified)

K)

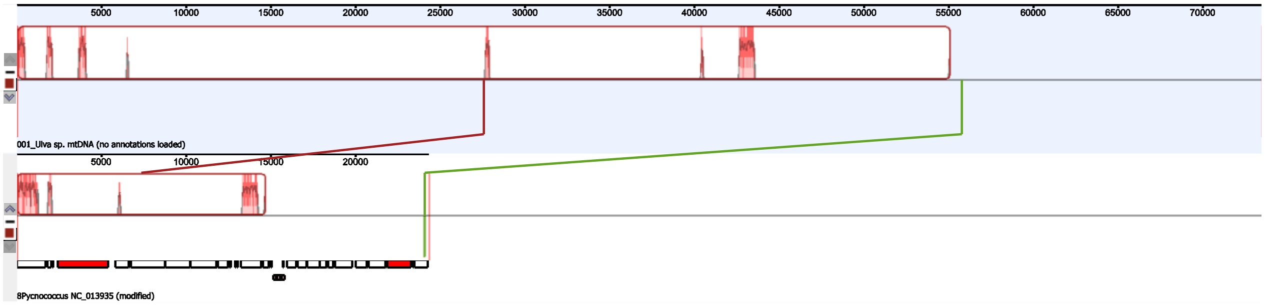
5000
10000
15000
20000
25000
30000
35000
40000
45000
50000
55000
60000
65000
70000
001_Ulva sp. mtDNA (no annotations loaded)
8Pycnococcus NC_013935 (modified)

L)
