# Supplementary material for: The Complete Chloroplast and Mitochondrial Genomes of the Green Macroalga Ulva sp. UNA00071828 (Ulvophyceae, Chlorophyta)
Source: PLoS One. 2015 Apr 7;10(4):e0121020. doi: 10.1371/journal.pone.0121020 (PMC4388391; doi:10.1371/journal.pone.0121020)
Supplement: S1 Table — (PDF) [file pone.0121020.s010.pdf]

S1 Table. GenBank accession numbers used in the *rbc*L and *tuf*A phylogenetic trees.

**Taxon**	***rbc*L**	***tuf*A**
Ulva compressa	AF387106	JN029296
Ulva fasciata	EU933962	JN029306
Ulva flexuosa	EF110051	JN029309
Ulva intestinalis	EU933937	JN029320
Ulva lactuca	AY422546	HQ610326
Ulva linza	AB741533	EF595300
Ulva meridionalis	AB598813	-
Ulva prolifera	-	HQ610403
Ulva rigida	EU484395	HE600182
Ulva sp. OTU1	GU138253	-
Ulva sp. OTU6	GU138251	-
Ulva sp OTU11	GU138249	-
Ulva sp.	AB598814	-
Blidingia minima	AF387109	HQ610329
Monostroma grevillei	GU183089	-
Monostroma sp.	-	HQ610262
