# Supplementary material for: The Complete Chloroplast and Mitochondrial Genomes of the Green Macroalga Ulva sp. UNA00071828 (Ulvophyceae, Chlorophyta)
Source: PLoS One. 2015 Apr 7;10(4):e0121020. doi: 10.1371/journal.pone.0121020 (PMC4388391; doi:10.1371/journal.pone.0121020)
Supplement: S2 Table — (PDF) [file pone.0121020.s011.pdf]

**S2 Table.** ***Ulva*** **sp. chloroplast tRNAs compared with** ***Oltmannsiellopsis viridis*** **and** ***Pseudendoclonium akinetum*** **tRNAs.**

| | total tRNA genes | *trn*A(UGC) | *trn*C(GCA) | *trn*D(GUC) | *trn*E(UUC) | *trn*F(GAA) | *trn*F(AAA) | *trn*G(UUC) | *trn*G(GCC) | *trn*H(GUG) | *trn*I(CAU) | *trn*I(GAU) | *trn*K(UUU) | *trn*L(UAG) | *trn*L(UAA) | *trn*L(CAA) | *trn*Me(CAU) | *trn*Mf(CAU) | *trn*N(GUU) | *trn*N(AUU) | *trn*N(GUU) | *trn*P(UGG) | *trn*Q(UGG) | *trn*R(UCU) | *trn*R(ACG) | *trn*R(CCU) | *trn*S(GCU) | *trn*R(CCG) | *trn*S(UGA) | *trn*S(UGA) | *trn*T(UGU) | *trn*V(UAC) | *trn*W(CCA) | *trn*Y(GUA) |
|---|---|---|---|---|---|---|---|---|---|---|---|---|---|---|---|---|---|---|---|---|---|---|---|---|---|---|---|---|---|---|---|---|---|---|
| *Ulva* sp. | 28 | 1 | 1 | 1 | 1 | 1 | 1 | 1 | 1 | 1 | 1 | 1 | 1 | 1 | 1 | 0 | 1 | 1 | 1 | 1 | 0 | 1 | 1 | 1 | 1 | 0 | 1 | 0 | 1 | 0 | 1 | 1 | 1 | 1 |
| *O. viridis* | 28 | 2 | 1 | 1 | 1 | 1 | 0 | 1 | 1 | 1 | 0 | 2 | 1 | 1 | 1 | 0 | 1 | 1 | 1 | 0 | 0 | 1 | 1 | 1 | 1 | 1 | 1 | 0 | 1 | 0 | 1 | 1 | 1 | 1 |
| *P. akinetum* | 31 | 2 | 1 | 1 | 1 | 1 | 0 | 1 | 1 | 1 | 1 | 2 | 1 | 1 | 1 | 1 | 1 | 1 | 1 | 0 | 1 | 1 | 1 | 1 | 1 | 1 | 0 | 1 | 1 | 1 | 1 | 1 | 1 | 1 |
