# Supplementary material for: The Complete Chloroplast and Mitochondrial Genomes of the Green Macroalga Ulva sp. UNA00071828 (Ulvophyceae, Chlorophyta)
Source: PLoS One. 2015 Apr 7;10(4):e0121020. doi: 10.1371/journal.pone.0121020 (PMC4388391; doi:10.1371/journal.pone.0121020)
Supplement: S3 Table — (PDF) [file pone.0121020.s012.pdf]

S3 Table. ***Ulva*** **sp. mitochondrial tRNAs compared with** ***Oltmannsiellopsis viridis*** **and** ***Pseudendoclonium akinetum*** **tRNAs.**

	total tRNA genes	*trn*A(UGC)	*trn*C(GCA)	*trn*D(GUC)	*trn*D(GUG)	*trn*E(UUC)	*trn*F(GAA)	*trn*G(GCC)	*trn*G(UCC)	*trn*H(GUG)	*trn*I(CAU)	*trn*I(GAU)	*trn*K(UUU)	*trn*L(UAA)	*trn*L(UAG)	*trn*M(CAU)	*trn*N(GUU)	*trn*P(UGG)	*trn*Q(UUG)	*trn*R(ACG)	*trn*R(UCU)	*trn*R(GCG)	*trn*R(UCG)	*trn*S(GCU)	*trn*S(UGA)	*trn*T(UGU)	*trn*V(UAC)	*trn*W(CCA)	*trn*Y(GUA)
Ulva sp.	26	1	1	0	1	1	1	0	1	1	0	1	1	1	1	3	1	1	1	0	1	1	1	0	1	1	1	1	1
O. viridis	24	1	1	1	0	1	1	1	1	1	1	0	1	1	1	2	1	1	1	1	1	0	0	1	1	0	1	1	1
P. akinetum	25	1	1	1	0	1	1	0	1	1	1	1	1	1	1	2	1	1	1	0	1	0	1	1	1	1	1	1	1
