# Supplementary material for: The Complete Chloroplast and Mitochondrial Genomes of the Green Macroalga Ulva sp. UNA00071828 (Ulvophyceae, Chlorophyta)
Source: PLoS One. 2015 Apr 7;10(4):e0121020. doi: 10.1371/journal.pone.0121020 (PMC4388391; doi:10.1371/journal.pone.0121020)
Supplement: S4 Table — (PDF) [file pone.0121020.s013.pdf]

	Ulva	*Pseudendoclonium*	*Oltmannsiellopsis*	*Bryopsis*	*Chlorella*	*Coccomyxa*	*Acutodesmus*	*Dunaliella*	*Oedogonium*	*Pedinomonas*	*Ostreococcus*	*Pycnococcus*
Ulva	0	22	36	40	36	43	54	52	51	34	34	35
Pseudendoclonium	-	0	38	41	36	44	49	52	55	35	34	34
Oltmannsiellopsis	-	-	0	42	30	40	55	55	57	31	31	31
Bryopsis	-	-	-	0	44	47	57	52	57	44	37	39
Chlorella	-	-	-	-	0	36	52	51	56	24	31	32
Coccomyxa	-	-	-	-	-	0	52	51	58	36	32	35
Acutodesmus	-	-	-	-	-	-	0	35	54	49	41	49
Dunaliella	-	-	-	-	-	-	-	0	52	49	38	50
Oedogonium	-	-	-	-	-	-	-	-	0	52	45	52
Pedinomonas	-	-	-	-	-	-	-	-	-	0	30	32
Ostreococcus	-	-	-	-	-	-	-	-	-	-	0	30
Pycnococcus	-	-	-	-	-	-	-	-	-	-	-	0

S4 Table. DCJ values calculated by UniMoG of chlorophyte cpDNAs without tRNAs.
