# Supplementary material for: The Complete Chloroplast and Mitochondrial Genomes of the Green Macroalga Ulva sp. UNA00071828 (Ulvophyceae, Chlorophyta)
Source: PLoS One. 2015 Apr 7;10(4):e0121020. doi: 10.1371/journal.pone.0121020 (PMC4388391; doi:10.1371/journal.pone.0121020)
Supplement: S5 Table — (PDF) [file pone.0121020.s014.pdf]

| | *Ulva* | *Pseudendoclonium* | *Oltmannsiellopsis* | *Prototheca* | *Helicosporidium* | *Chlorella* | *Acutodesmus* | *Pedinomonas* | *Nephroselmis* | *Ostreococcus* | *Pycnococcus* |
|---|---|---|---|---|---|---|---|---|---|---|---|
| *Ulva* | 0 | 17 | 25 | 25 | 26 | 25 | 11 | 10 | 24 | 26 | 14 |
| *Pseudendoclonium* | - | 0 | 27 | 24 | 27 | 25 | 11 | 12 | 25 | 24 | 17 |
| *Oltmannsiellopsis* | - | - | 0 | 27 | 26 | 24 | 10 | 10 | 25 | 20 | 16 |
| *Prototheca* | - | - | - | 0 | 15 | 15 | 12 | 9 | 27 | 20 | 14 |
| *Helicosporidium* | - | - | - | - | 0 | 12 | 9 | 11 | 28 | 21 | 14 |
| *Chlorella* | - | - | - | - | - | 0 | 10 | 9 | 24 | 19 | 14 |
| *Acutodesmus* | - | - | - | - | - | - | 0 | 6 | 10 | 10 | 11 |
| *Pedinomonas* | - | - | - | - | - | - | - | 0 | 10 | 5 | 8 |
| *Nephroselmis* | - | - | - | - | - | - | - | - | 0 | 12 | 16 |
| *Ostreococcus* | - | - | - | - | - | - | - | - | - | 0 | 11 |
| *Pycnococcus* | - | - | - | - | - | - | - | - | - | - | 0 |

**S5 Table. DCJ values calculated by UniMoG of chlorophyte mtDNAs without tRNAs.**
