# Supplementary material for: The Complete Chloroplast and Mitochondrial Genomes of the Green Macroalga Ulva sp. UNA00071828 (Ulvophyceae, Chlorophyta)
Source: PLoS One. 2015 Apr 7;10(4):e0121020. doi: 10.1371/journal.pone.0121020 (PMC4388391; doi:10.1371/journal.pone.0121020)
Supplement: S6 Table — (PDF) [file pone.0121020.s015.pdf]

S6 Table. GenBank accession numbers of the taxon sampling for phylogenomics.

Acetabularia acetabulum	HG518425-HG794360
Acutodesmus obliquus	NC_008101
Botryococcus braunii	KM464687-KM504519
Bryopsis hypnoides	NC_013359
Cephaleuros parasiticus	KM464687-KM504519
Chlamydomonas reinhardtii	NC_005353
Chlorella vulgaris	NC_001865
Coccomyxa subellipsoidea C-169	NC_015084
Codium decorticatum	KM820109-KM820167
Dunaliella salina	NC_016732
Elliptochloris bilobata	KM462887
Floydiella terrestris	NC_014346
Geminella minor	KM462883
Halimeda cylindracea	KM820107-KM820166
Hemichloris antarctica	HQ317295-KM491867
Koliella corcontica	KM462874
Leptosira terrestris	NC_009681
Lobosphaera incisa	KM462871
Marsupiomonas sp. NIES 1824	KM462870
Marvania geminata	KM462888
Microthamnion kuetzingianum	KM462876
Monomastix sp. OKE-1	NC_012101
Neocystis brevis	KM462873
Nephroselmis astigmatica	KJ746600
Nephroselmis olivacea	NC_000927
Oedogonium cardiacum	NC_011031
Oltmannsiellopsis viridis	NC_008099
Oocystis solitaria	FJ968739
Ostreococcus tauri	NC_008289
Pabia signiensis	KM462866
Parachlorella kessleri	NC_012978
Pedinomonas minor	NC_016733
Pedinomonas tuberculata	KM462867
Picocystis salinarum	NC_024828
Prasinococcus sp. CCMP1194	KJ746597
Prasinoderma coloniale	NC_024817
Prasinophyceae sp. CCMP1205	KJ746601

Prasinophyceae sp. MBIC10622	KJ746602
Prasiolopsis sp. SAG 84.81	KM462862
Pseudendoclonium akinetum	NC_008114
Pseudochloris wilhelmii	KM462886
Pycnococcus provasolii	NC_012097
Pyramimonas parkeae	NC_012099
Schizomeris leibleinii	HQ700713
Stigeoclonium helveticum	NC_008372
Tetraselmis sp.	AB561008-AB561081, DQ173248, DQ173249, DQ227304, HF931099
Trebouxia aggregata	EU123963-EU124002
Trentepohlia annulata	KM464689-KM491845
Ulva sp.	KP720616
Volvox carteri f. nagariensis	GU084820
Watanabea reniformis	KM462863
Xylochloris irregularis	KM462872
