# Supplementary material for: The Complete Chloroplast and Mitochondrial Genomes of the Green Macroalga Ulva sp. UNA00071828 (Ulvophyceae, Chlorophyta)
Source: PLoS One. 2015 Apr 7;10(4):e0121020. doi: 10.1371/journal.pone.0121020 (PMC4388391; doi:10.1371/journal.pone.0121020)
Supplement: S9 Fig — (PDF) [file pone.0121020.s009.pdf]

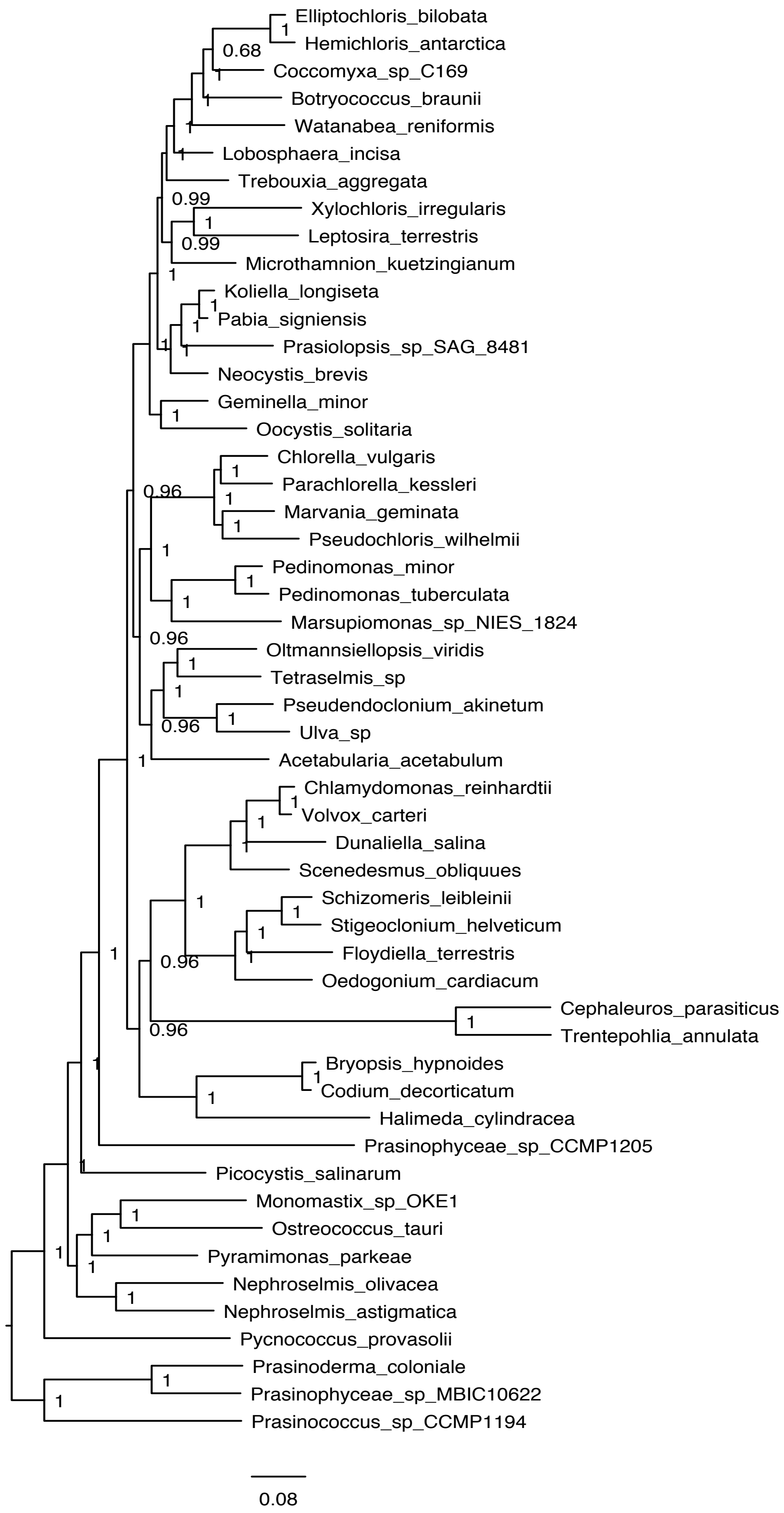

S9 Fig. Bayesian tree with posterior probabilities based on AA alignment of 51 chlorophyte genes.
